# UBIAD1 alleviates ferroptotic neuronal death by enhancing antioxidative capacity by cooperatively restoring impaired mitochondria and Golgi apparatus upon cerebral ischemic/reperfusion insult

**DOI:** 10.1186/s13578-022-00776-9

**Published:** 2022-04-04

**Authors:** Yan Huang, Jianyang Liu, Jialin He, Zhiping Hu, Fengbo Tan, Xuelin Zhu, Fulai Yuan, Zheng Jiang

**Affiliations:** 1 NHC Key Laboratory of Birth Defect for Research and Prevention (Hunan Provincial Maternal and Child Health Care Hospital), Changsha, Hunan 410008 People’s Republic of China; 2 Hunan Provincial Maternal and Child Health Care Hospital, Changsha, Hunan 410008 People’s Republic of China; 3 Hunan Provincial Key Laboratory of Neurorestoration, Hunan Normal University, Changsha, Hunan 410081 People’s Republic of China; 4 Department of Neurology, The Second Xiangya Hospital, Central South University, 139 Renming Road, Changsha, Hunan 410011 People’s Republic of China; 5 Department of Gastrointestinal Surgery, Xiangya Hospital, Central South University, Changsha, Hunan 410008 People’s Republic of China; 6 Department of Radiology, Xiangya Hospital, Central South University, Changsha, Hunan 410008 People’s Republic of China; 7 Health Management Center, Xiangya Hospital, Central South University, Changsha, Hunan 410008 People’s Republic of China; 8 National Clinical Research Center for Geriatric Disorders, Xiangya Hospital, Central South University, Changsha, Hunan 410008 People’s Republic of China

**Keywords:** UBIAD1, Ferroptotic neuronal death, Mitochondria, Golgi apparatus, Cerebral ischemia/reperfusion

## Abstract

**Background:**

Neuronal death due to over-oxidative stress responses defines the pathology of cerebral ischemic/reperfusion (I/R) insult. Ferroptosis is a form of oxidative cell death that is induced by disruption of the balance between antioxidants and pro-oxidants in cells. However, the potential mechanisms responsible for cerebral I/R-induced ferroptotic neuronal death have not been conclusively determined. UBIAD1, is a newly identified antioxidant enzyme that catalyzes coenzyme Q10 (CoQ10) and vitamin K2 biosynthesis in the Golgi apparatus membrane and mitochondria, respectively. Even though UBIAD1 is a significant mediator of apoptosis in cerebral I/R challenge, its roles in ferroptotic neuronal death remain undefined. Therefore, we investigated whether ferroptotic neuronal death is involved in cerebral I/R injury. Further, we evaluated the functions and possible mechanisms of UBIAD1 in cerebral I/R-induced ferroptotic neuronal death, with a major focus on mitochondrial and Golgi apparatus dysfunctions.

**Results:**

Ferroptosis occurred in cerebral I/R. Ferroptotic neuronal death promoted cerebral I/R-induced brain tissue injury and neuronal impairment. UBIAD1 was expressed in cerebral tissues and was localized in neurons, astrocytes, and microglia. Under cerebral I/R conditions overexpressed UBIAD1 significantly suppressed lipid peroxidation and ferroptosis. Moreover, upregulated UBIAD1 protected against brain tissue damage and neuronal death by alleviating I/R-mediated lipid peroxidation and ferroptosis. However, UBIAD1 knockdown reversed these changes. Enhanced UBIAD1-mediated ferroptosis elevated the antioxidative capacity by rescuing mitochondrial and Golgi apparatus dysfunction in cerebral I/R-mediated neuronal injury. They improved the morphology and biofunctions of the mitochondria and Golgi apparatus, thereby elevating the levels of SOD, T-AOC and production of CoQ10, endothelial nitric oxide synthase (eNOS)-regulated nitric oxide (NO) generation as well as suppressed MDA generation.

**Conclusions:**

The neuroprotective agent, UBIAD1, modulates I/R-mediated ferroptosis by restoring mitochondrial and Golgi apparatus dysfunction in damaged brain tissues and neurons, thereby enhancing antioxidative capacities. Moreover, the rescue of impaired mitochondrial and Golgi apparatus as a possible mechanism of regulating ferroptotic neuronal death is a potential treatment strategy for ischemic stroke.

**Graphical Abstract:**

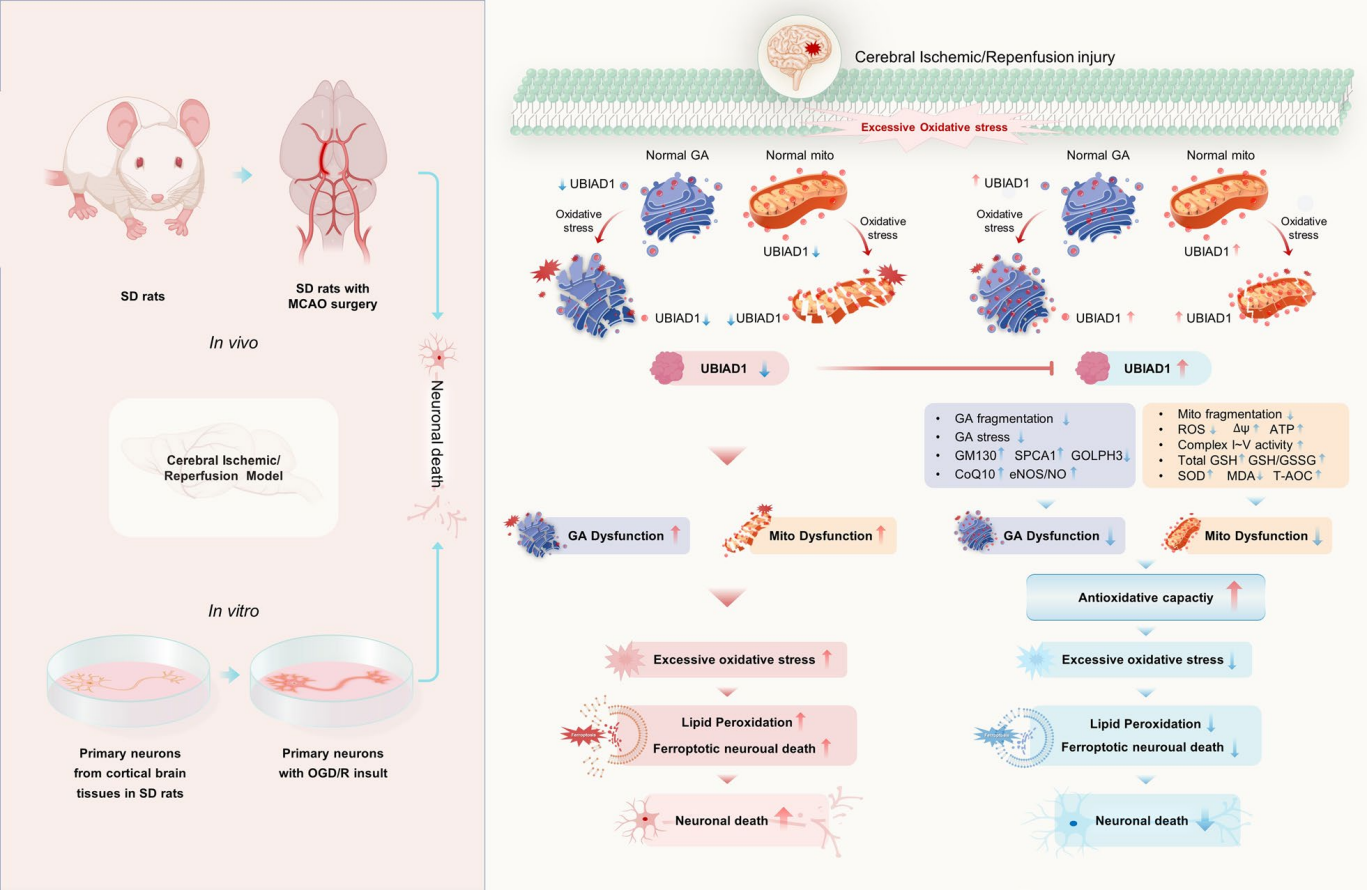

**Supplementary Information:**

The online version contains supplementary material available at 10.1186/s13578-022-00776-9.

## Introduction

Cerebral ischemic stroke is a huge threat to human health. Currently, prompt thrombolytic therapy remains the mainstay clinical therapeutic option for ischemic stroke [1, 2]. Although the interruption of cerebral blood supply can be restored via thrombolytic treatment, reperfusion might aggravate ischemia brain tissue injury, resulting in cerebral ischemia/reperfusion (I/R) injuries [3, 4]. Secondary insult from cerebral I/R damage is associated with neuronal cell death or damage to brain regions, leading to impaired cognitive functions, disability, and even death [5, 6]. Therefore, it is important to establish novel strategies to alleviate cerebral I/R impairment-induced neuronal death.

Cellular death due to excess oxidative stress responses characterizes the pathology of various neurological disorders, including cerebral I/R-induced neuronal death [7, 8]. However, the potential mechanisms underlying cerebral I/R-induced oxidative neuronal death have not yet been established. Recently, ferroptosis has been shown to be a form of oxidative programmed cell death [9]. Ferroptosis is caused by the accumulation of iron-dependent lipid peroxidation coupled with reduction of glutathione (GSH), which overwhelm the cells’ antioxidant defense system and eventually contributes to oxidative cellular death [10]. The ferroptosis-associated oxidative cell death is often common in neuronal death provoked by ischemic stroke [11]. Moreover, ferroptosis mediates the actions of cerebral I/R [12]. Therefore, modulation of ferroptotic neuronal death might be a potential treatment strategy for cerebral I/R injury. However, the mechanisms involved in ferroptotic neuronal death after cerebral I/R injury have not been conclusively determined.

Ferroptosis is characterized by imbalances between oxidation and antioxidation. Following ferroptosis, lipid peroxidation is the most crucial mechanism associated with plasma membrane insults [13]. There is a close interplay between excess accumulation of lipid peroxidation, and mitochondrial or Golgi apparatus dysfunctions [14]. Besides, typical morphological characteristics of ferroptosis include impairments of mitochondrial morphology and structure [15]. Mitochondrial insults mediate lipid peroxidation and ferroptosis through mitochondrial fragmentation, increased ROS production, and inhibition of mitochondrial metabolism [16, 17]. Jelinek et al. reported that rescuing mitochondrial damage prevented glutathione peroxidase-dependent ferroptosis and accumulation of lipid peroxidation in neuronal HT22 cells [18]. Thus, mitochondrial dysfunction might aggravate lipid peroxidation and ferroptosis via excess oxidative stress, leading to irreversible cell death, including cerebral I/R induced neuronal death. Induction of Golgi apparatus stress by brefeldin A (BFA) contributed to over-activation of lipid peroxidation, which enhanced ferroptosis in human cell lines [19]. As early as 1995, Rafols et al. [20] reported on the accumulation of lipid peroxidation in Golgi apparatus upon cerebral I/R in rat models. Overexpression of small G protein (ARF1)-associated Golgi apparatus showed resistance to Golgi apparatus stress and lipid peroxidation [19]. The Golgi apparatus might be involved in regulation of oxidative stress, lipid peroxidation and ferroptosis. Therefore, it is important to identify possible neuroprotective mediators to rescue mitochondrial and Golgi apparatus dysfunctions.

UBIAD1, also referred to as TERE1, is a newly identified antioxidant enzyme that catalyzes CoQ10 biosynthesis in Golgi apparatus membranes [21]. In non-mitochondrial CoQ10 systems, CoQ10 is involved in regulation of lipid peroxidation generation, which acts in parallel to the glutathione peroxidase 4 (GPX4) pathway in ferroptosis [22]. Downregulation of UBIAD1 suppresses antioxidant capacities, which impairs lipid peroxidation [21]. Biologically, UBIAD1 is a critical biosynthetic enzyme of vitamin K2, which serves as an electron transporter in maintaining mitochondrial metabolism [23, 24]. Knock-down of UBIAD1 impaired mitochondrial morphology and metabolism in carcinoma cells lines [25]. Overexpressed UBIAD1 enhances excess generation of ROS/RNS and upregulation of mitochondrial membrane potential [21]. In addition, UBIAD1 is vital in modulation of cholesterol metabolism and HMG CoA reductase [26]. Due to UBIAD1 mutations, schnyder corneal dystrophy (SCD) induces the over production of cholesterol in the cornea [27]. Moreover, UBIAD1 inhibited apoptosis by ameliorating oxidatively insulted mitochondrial and Golgi apparatus under oxygen-glucose deprivation and reperfusion (OGD/R) conditions in N2A cells [28]. These findings imply that UBIAD1 is involved in inhibition of oxidative stress, lipid peroxidation and cholesterol metabolism by modulating mitochondrial and Golgi apparatus dysfunctions. However, the functions of UBIAD1 and its possible mechanisms in cerebral I/R insult associated with lipid peroxidation and ferroptotic neuronal death are undefined.

We hypothesized that UBIAD1 is involved in cerebral I/R damage-mediated lipid peroxidation and ferroptotic neuronal death. We found that inhibition of lipid peroxidation and ferroptotic neuronal death blocks cerebral I/R insults in brain tissues and neurons. Besides, overexpressed UBIAD1 improved brain tissue impairment and neuronal death induced by I/R-mediated ferroptosis. Biologically, UBIAD1 enhances antioxidative capacities by restoring impaired mitochondria and Golgi apparatus. This study provides a novel target and basis for treatment of cerebral ischemic stroke.

## Results

### Cerebral I/R insults induced ferroptotic neuronal death and lipid peroxidation in vivo and in vitro

**Fig. 1:**
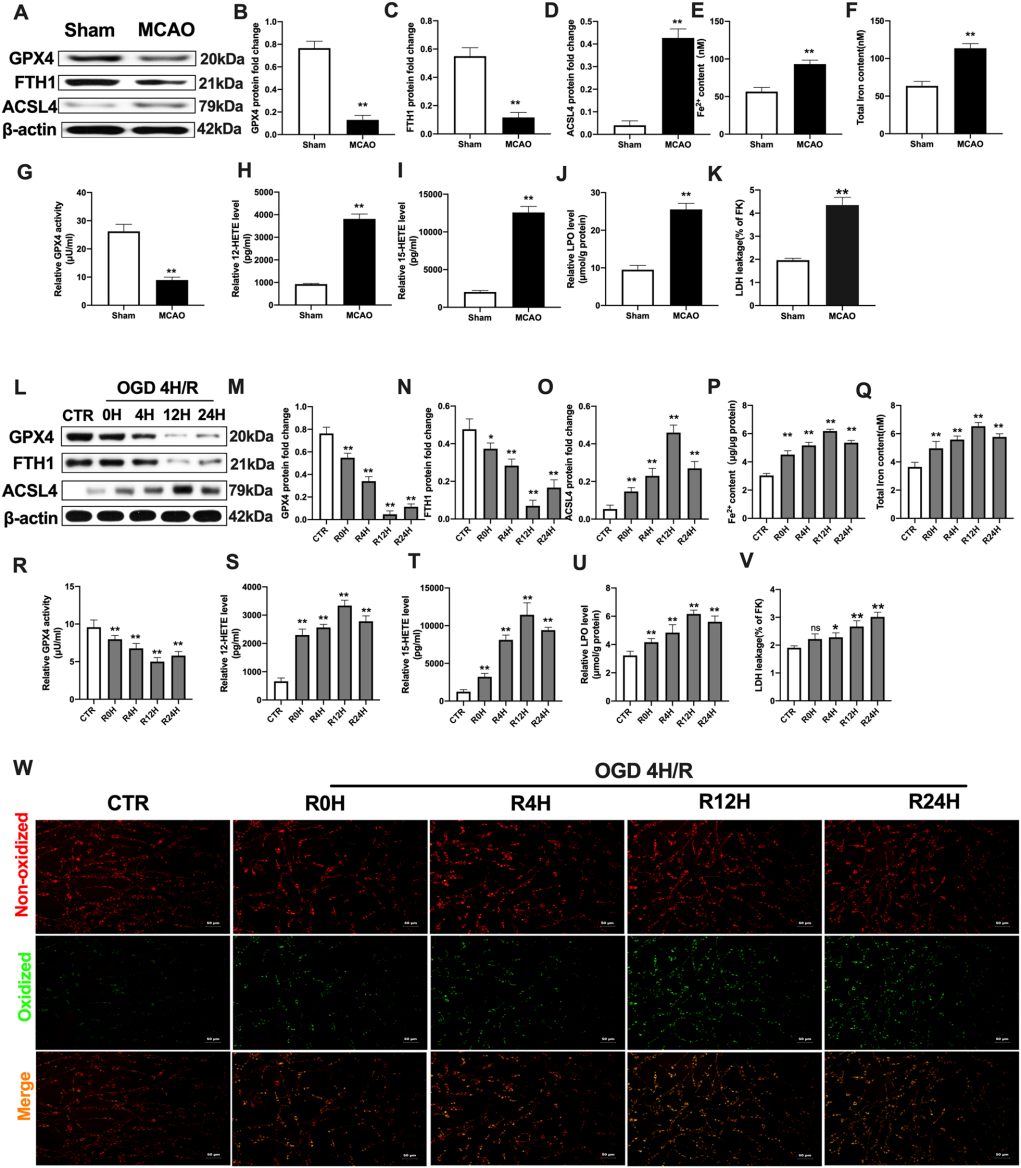
Cerebral I/R insult induced ferroptotic neuronal death and lipid peroxidation in vivo and vitro. SD rats were subjected to MCAO for 90 min and then reperfused to induce cerebral I/R insult in vivo. Peri-ischemic cortex brain tissues were evaluated after 14 days of reperfusion. **A**–**D** The protein expression of GPX4, FTH1, and ASCL4 after MCAO as determined by western blotting. **E**, **F** The content of Fe^2+^ and total iron as measured with the iron assay kit. **G** GPX4 activity as assayed using GPX4 activity assay kit. **H**, **I** The levels of 12-HETE and 15-HETE as quantified using ELISA kits. **J** The level of LPO as assayed using lipid peroxidation assay kit. **K** The level of LDH as quantified using LDH assay kit. Primary neurons were exposed to OGD/R to induce cerebral I/R insult (OGD: 4 h, Reperfusion: 0-, 4-, 12-, 24-h). **L**–**O** The protein expression of GPX4, FTH1, and ASCL4 after OGD/R as determined by western blotting. **P**, **Q** The content of Fe^2+^ and total iron after OGD/R as assayed with the iron assay kit. **R** GPX4 activity after OGD/R as determined with GPX4 activity assay kit. **S**, **T** The levels of 12-HETE and 15-HETE after OGD/R as detected with ELISA kits. **U** The level of LPO after OGD/R as assayed using lipid peroxidation assay kit. **V** The level of LDH after OGD/R as determined using LDH assay kit. **W** Lipid peroxidation in neurons as assayed with the BODIPY 581/591 C11 staining assay (Scale bar = 50 μm). n = 3 for in vitro tests, n = 6 rats/group for in vivo tests. All data are expressed as the mean ± SD, *P < 0.05, **P < 0.01; relative to the sham group and control group

To characterize the occurrence of ferroptotic neuronal death and lipid peroxidation in cerebral I/R challenge, we profiled ferroptosis-associated protein expressions and factors in rat brain tissues and cultured primary neurons, respectively. In vivo*,* the expressions of negative mediators (GPX4 and Ferritin1, FTH1) [29, 30] were significantly suppressed in the MCAO group, compared to the sham group in rat brain tissues (Fig. 1A–C). On the contrary, levels of the positive mediator of Acyl-CoA synthetase long-chain family member 4 (ACSL4) [30, 31] were elevated in MCAO group, relative to the sham group (Fig. 1A, D). Then, we evaluated the changes in iron levels after MCAO insult. Compared to the sham group, Fe^2+^ and total iron levels were markedly increased in the MCAO group (Fig. 1E, F). With regards to alterations of lipid peroxidation-related factors [30] upon MCAO insult, there was the suppression of GPX4 activities in the MCAO group, compared with the sham group, while 12-HETE and 15-HETE levels were elevated in the MCAO group (Fig. 1G–I). In addition, levels of lipid peroxidase (LPO) and lactate dehydrogenase (LDH) were markedly augmented after MCAO insults in rat brain tissues, compared to the sham group (Fig. 1J, K). These findings imply cerebral I/R-induced alterations in ferroptosis and lipid peroxidation-associated factors in injured rat brain tissues.

To confirm the above in vivo results, an OGD/R in vitro model was established to assess the occurrence of ferroptosis in primary cortical neurons. Compared to the control group, GPX4 and FTH1 protein levels were markedly downregulated after OGD insults, and the suppression aligned with increasing reperfusion time point duration (Fig. 1L–N). At 12 h of reperfusion, GPX4 and FTH1 protein levels were lowest. In contrast, ACSL4 levels were elevated with prolonged reperfusion time point, which achieved peak values at 12 h of reperfusion following OGD injury, relative to the control group (Fig. 1L, O). Meanwhile, both FE^2+^ and total iron levels were remarkably higher in OGD/R-induced neurons, compared to the control group (Fig. 1P, Q). The activities of GPX4 were significantly inhibited in OGD plus different reperfusion time point group, relative to the control group in primary cortical neurons (Fig. 1R). Next, we evaluated the generation of lipid peroxidative-linked indices in neurons. As shown in Fig. 1S–U, compared to the control group, damaged primary neurons by OGD/R insults exhibited elevated lipid peroxidation levels, including significant enhancements of 12-HETE, 15-HETE and LPO levels. This showed enhanced lipid peroxidation due to OGD/R-induced neuronal damages. Further, the LDH level was markedly enhanced in the OGD/R group, relative to the control group (Fig. 1V). Besides, there was a significant increase in oxidized green fluorescence (via BODIPY581/591 C11 dye staining) in the OGD/R group, compared to the control group (Fig. 1W).

In conclusion, cerebral I/R induced the over-activation of lipid peroxidation and ferroptosis, indicating that ferroptotic neuronal death might be involved in cerebral I/R-induced insult.

### Suppression of ferroptosis and lipid peroxidation rescued cerebral I/R-induced injury in vivo and in vitro

**Fig. 2:**
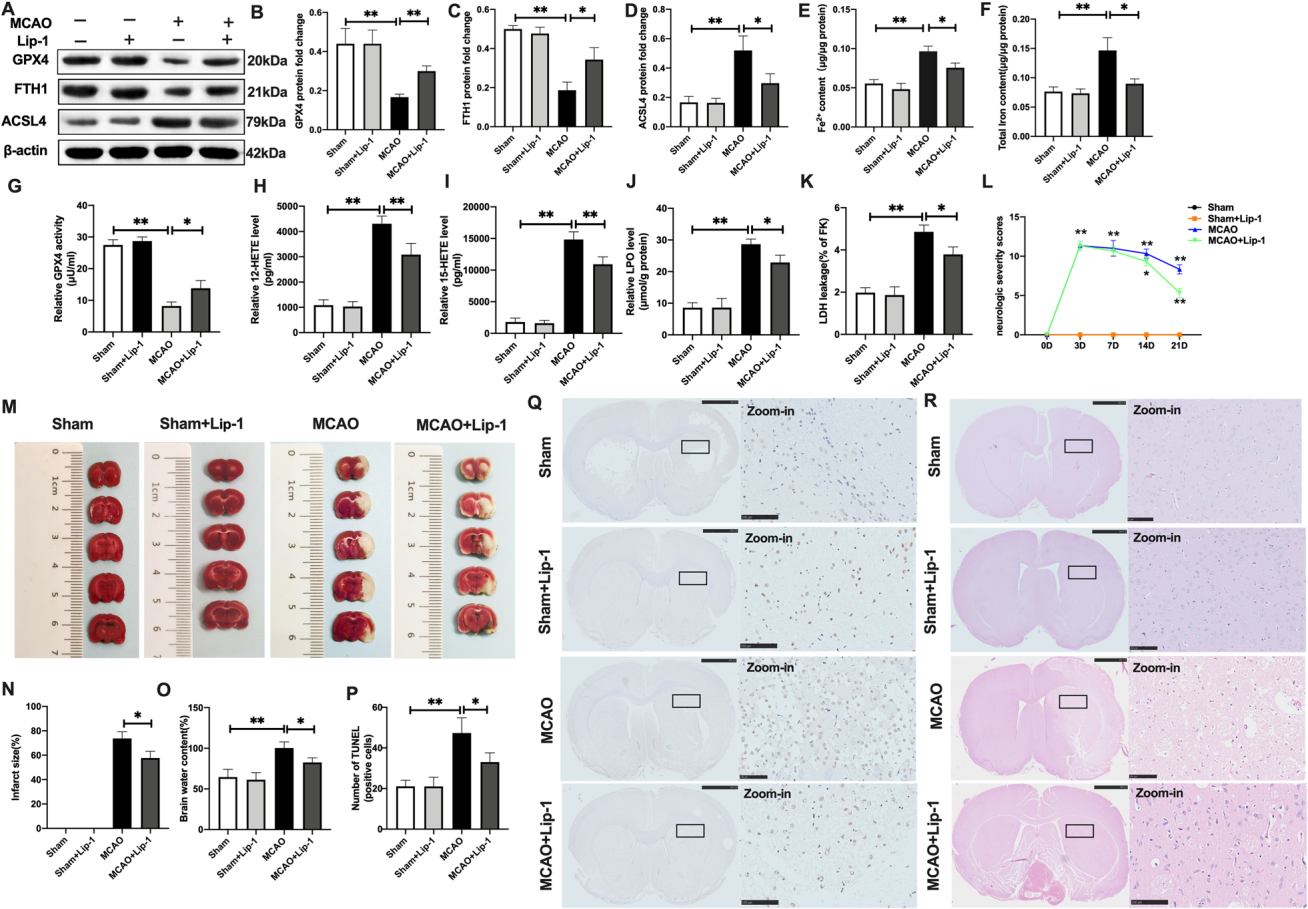
Suppression of ferroptosis and lipid peroxidation rescued cerebral I/R-induced brain injury in vivo. SD rats were treated with Lip-1 (i.p, 10 mg/kg) for 1 h and then subjected to MCAO operation. **A**–**D** The protein expression of GPX4, FTH1, and ASCL4 as assayed with western blotting. **E**, **F** Content of Fe^2+^ and total iron as determined with the iron assay kit. **G** GPX4 activity as detected using GPX4 activity assay kit. **H**, **I** The levels of 12-HETE and 15-HETE as determined with ELISA kits. **J** LPO level as assayed with the lipid peroxidation assay kit. **K** LDH level as quantified using LDH assay kit. **L** Neurological severity scores. **M**, **N** Infarct size in whole brain tissues as measured with TTC staining. **O** Brain water content. **P** TUNEL staining of rat brain tissues. **Q** HE staining of rat brain tissues (Scale bar = 50 μm). n = 6 rats/group, all data are expressed as the mean ± SD, *P < 0.05, **P < 0.01; MCAO group vs. the sham group and MCAO group vs. MCAO + Lip-1 group

To confirm ferroptotic neuronal death in cerebral I/R-induced damage, we evaluated the effects of suppression of ferroptotic neuronal death and lipid peroxidation in brain tissue injury. Liproxstain-1 (Lip-1), a ferroptosis suppressor [29], was used in the MCAO and OGD/R models. Compared to the MCAO group, GPX4 and FTH1 protein levels were significantly upregulated in rat brain tissues after Lip-1 treatment in MCAO + Lip-1 group (Fig. 2A–C). Comparatively, ACSL4 protein levels were inhibited by Lip-1 in the MCAO + Lip-1 group, relative to the MCAO group (Fig. 2A, D). However, differences in these protein levels between the sham and the sham + Lip-1 treatment groups were insignificant (Fig. 2A–D). In addition, compared to the MCAO group, ferroptosis suppression via Lip-1 significantly inhibited Fe^2+^ and total iron levels in the MCAO + Lip-1 group (Fig. 2E, F). Further, GPX4 activities were markedly improved in the MCAO + Lip-1 group, relative to the MCAO group (Fig. 2G). Meanwhile, levels of lipid peroxidation-associated indices [30], including 12-HETE and 15-HETE were markedly attenuated following MCAO + Lip-1 treatment, relative to the MCAO group (Fig. 2H, I). In addition, Lip-1 suppressed lipid peroxidation as exhibited by reduction of LPO and LDH levels in MCAO + Lip-1 group, compared to the naïve MCAO group (Fig. 2J, K). These findings imply that MCAO-induced ferroptosis and excess lipid peroxidation in injured brain tissues could be rescued by Lip-1 treatment, a ferroptosis inhibitor. Next, we evaluated the effects of Lip-1 on cerebral I/R-induced brain tissue insults. Differences in neurologic severity scores at 3- and 7-days in the post MCAO operation group and MCAO + Lip-1 group were insignificant (Fig. 2L). Neurologic severity scores were markedly low at 14- and 21-days in the MCAO + Lip-1 group, relative to the MCAO group (Fig. 2L). In addition, TTC staining revealed that compared to the MCAO group, the MCAO + Lip-1 group exhibited significant reductions in infarct sizes of rat brain tissues at 14 days, and a high decline in brain water contents (Fig. 2M–O). We performed HE and TUNEL staining to assess damaged brain tissues upon MCAO operation. Compared to the MCAO operation group, Lip-1 improved brain tissue injury and reduced neuronal death in the MCAO + Lip-1 group (Fig. 2P–R).

**Fig. 3:**
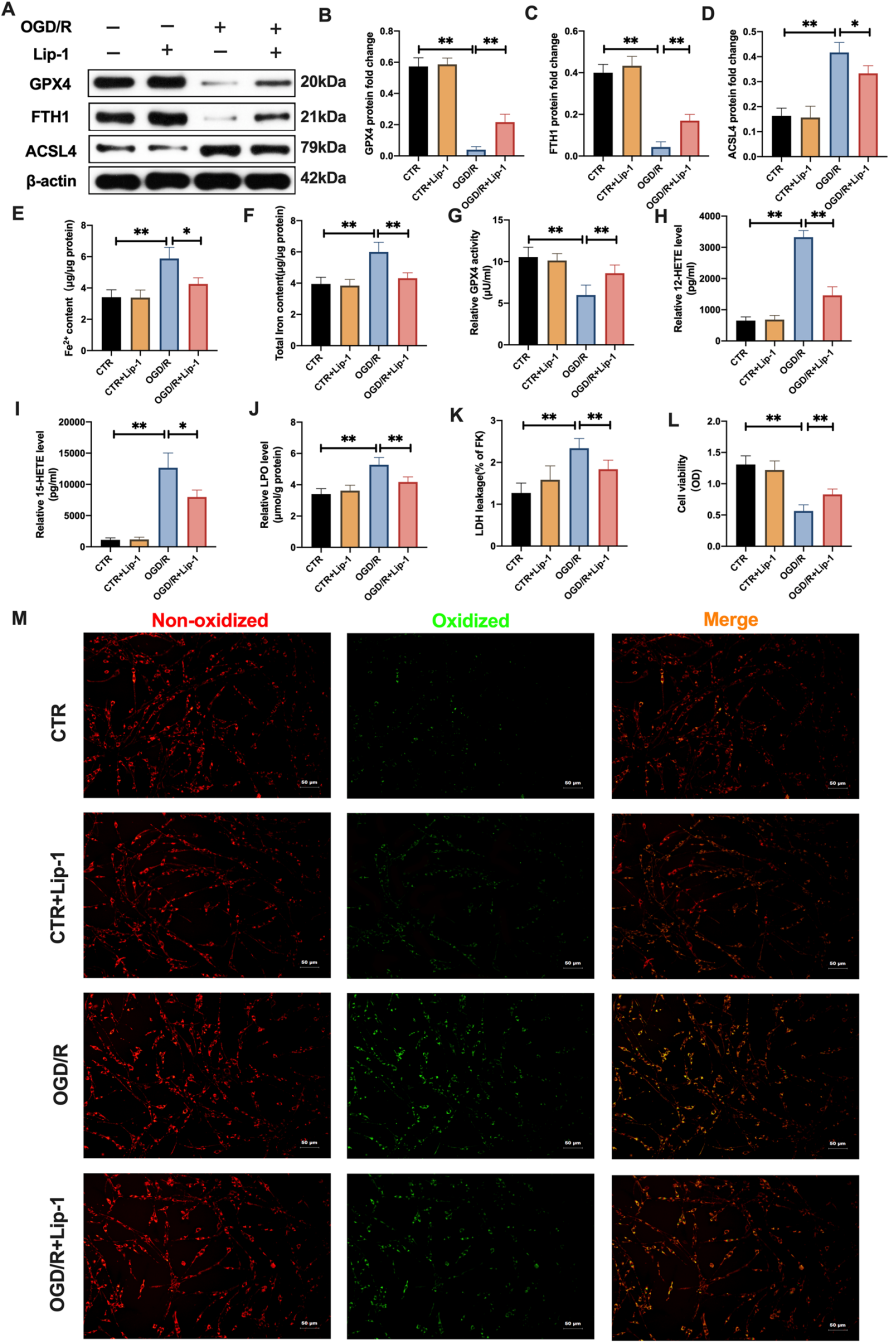
Inhibition of ferroptotic neuronal death and lipid peroxidation mitigated OGD/R-induced neuronal injury in vitro. Primary neurons were pre-incubated with Lip-1 (200 nM, 12 h) before exposure to OGR/R (OGD: 4 h, Reperfusion: 12 h). **A**–**D** The protein expression of GPX4, FTH1, and ASCL4 as quantified using western blotting assay. **E**, **F** Content of Fe^2+^ and total iron as determined with the iron assay kit. **G** GPX4 activity as determined with the GPX4 activity assay kit. **H**, **I** Levels of 12-HETE and 15-HETE as revealed by ELISA assay kits. **J** LPO level based on lipid peroxidation assay. **K** LDH level as evaluated using LDH assay kit. **L** Results of MTT assay showing cell viability. **M** Lipid peroxidation level in neurons as determined by the BODIPY 581/591 C11 staining (Scale bar = 50 μm). n = 3, all data are expressed as the mean ± SD, *P < 0.05, **P < 0.01; OGD/R group vs. CTR group and OGD/R group vs. OGD/+Lip-1 group

Primary neurons were cultured with Lip-1 for 12 h after which they were subjected to OGD/R pressure. After OGD insults for 4 h, there were alterations in ferroptosis-associated indices at 12 h of reperfusion. Therefore, we selected for OGD 4 h and reperfusion for 12 h for subsequent assays. As shown in Fig. 3A–D, in tandem with in vivo results, under OGD/R pressure, Lip-1 significantly restored GPX4 and FTH1 protein levels, and effectively suppressed ACSL4 protein levels. In addition, elevated Fe^2+^ and total iron levels were inhibited by Lip-1 treatment in the OGD/R + Lip-1 group, compared to the OGD/R group (Fig. 3E, F). Besides, Lip-1 suppression of GPX4 activities was significantly enhanced in the OGD/R + Lip-1 group, relative to the OGD/R group (Fig. 3G). A combination of Lip-1 with OGD/R insulted neurons markedly suppressed 12-HETE and 15-HETE levels, relative to the OGD/R group (Fig. 3H, I). Similarly, LPO and LDH levels were significantly suppressed in the OGD/R + Lip-1 group, compared to the OGD/R group (Fig. 3J, K). In addition, relative to the OGD/R group, increased green fluorescence stand for oxidizing neurons (BODIPY 581/591 C11 staining) were obviously inhibited by Lip-1 treatment with insulted neurons after OGD/R (Fig. 3M). Moreover, the viability of cell neurons was significantly elevated following Lip-1 coupled with OGD/R treatment, relative to the OGD/R group (Fig. 3L).

These findings imply the involvement of ferroptotic neuronal death in cerebral I/R damage and indicate that Lip-1 protects against cerebral I/R insult, rescues injured brain tissues and neuronal death by suppressing ferroptosis and lipid peroxidation.

### Expressions and localization of UBIAD1 in response to cerebral I/R injuries in vivo and in vitro

**Fig. 4:**
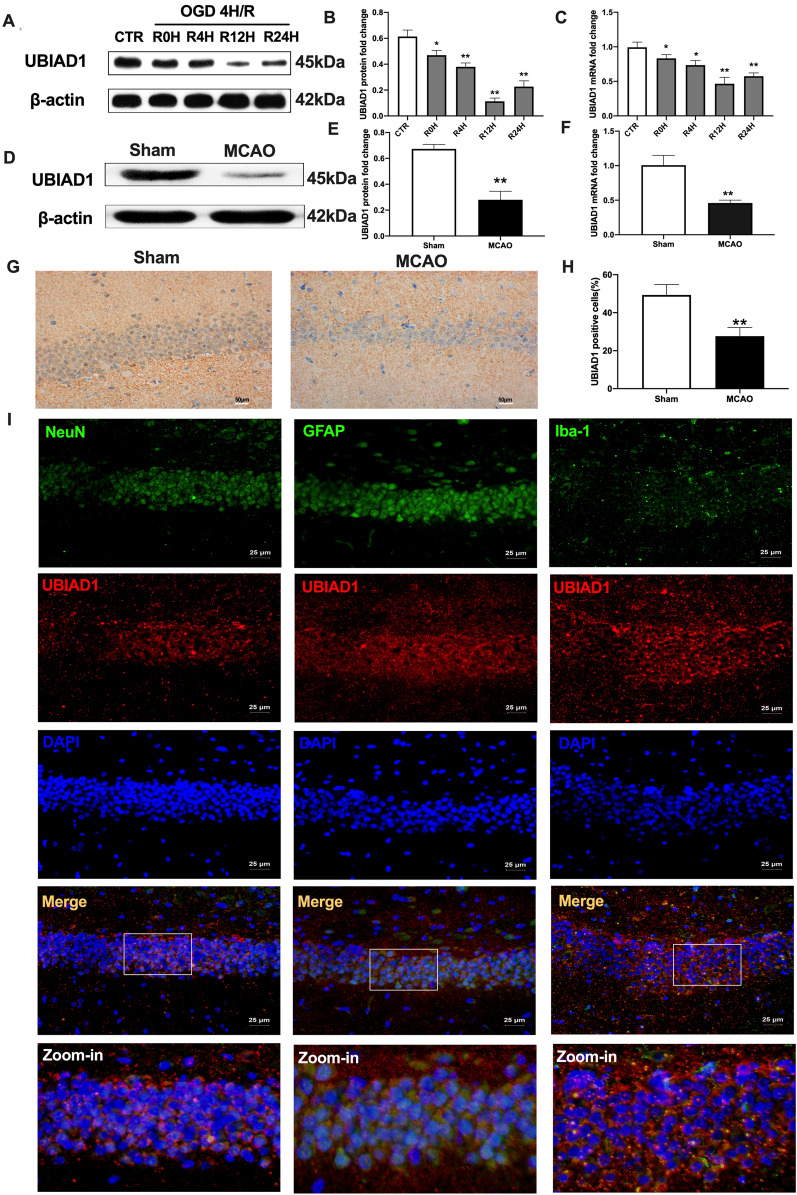
Expression and location of UBIAD1 in response to cerebral I/R injury in vivo and vitro. **A**–**C** The protein and mRNA expression of UBIAD1 in neurons as detected with western blotting and PCR assay. **D**–**F** The protein and mRNA expression of UBIAD1 in brain tissues as detected with western blotting and PCR assay. **G**, **H** Protein expression of UBIAD1 in brain tissues as evaluated through immunohistochemical staining (Scale bar = 50 μm). **I** Confocal images showing co-localization of UBIAD1 (red) with neuron marker NeuN (green), astrocytes marker GFAP (green) microglia marker Iba-1 (green) (Scale bar = 25 μm). n = 3 for in vitro, n = 6 rats/group for in vivo, all data are expressed as the mean ± SD, *P < 0.05, **P < 0.01; relative to the control group or sham group

To investigate UBIAD1 functions in cerebral I/R insult-induced ferroptotic neuronal death, we evaluated the expressions of UBIAD1 in MCAO and OGD/R conditions. In vitro, after OGD 4 h insults, gradual suppressions of UBIAD1 mRNA and protein levels were proportional to reperfusion time (Fig. 4A–C). Interestingly, UBIAD1 levels were least after 12 h of reperfusion (Fig. 4A–C). In vivo*,* compared to the sham group, UBIAD1 protein levels were markedly suppressed in rat brain tissues of the MCAO group (Fig. 4D–E). Consistent with western-blot findings, UBIAD1 mRNA levels were markedly suppressed after MCAO operation, relative to sham group (Fig. 4F). Immunohistochemistry showed that positive UBIAD1 levels in rat brain tissues were inhibited post MCAO, compared to sham group (Fig. 4G, H). Therefore, UBAID1 expressions were induced by cerebral I/R injury.

Having demonstrated the reduction of UBIAD1 expressions in impaired rat brain tissues as well as insulted primary neurons because of cerebral I/R, we profiled the co-localizations of principle cell types in brain tissues, such as neurons, astrocytes and microglia with UBIAD1. Using typical markers for neurons (NeuN) [32], astrocytes (GFAP) [33], microglia (Iba-1) [34] and UBIAD1 antibodies, we performed immunofluorescence assays in hippocampus regions of brain tissues. Double immunofluorescence revealed that UBIAD1 was highly expressed in all cell-types, including NeuN-positive cells, astrocytes- and microglia-positive cells (Fig. 4I).

These findings show the expressions of UBIAD1 in cerebral tissues, its localization in neurons, astrocytes, and microglia as well as its involvement in cerebral I/R insult.

### UBIAD1 regulated OGD/R-induced ferroptotic neuronal death and lipid peroxidation in vitro

**Fig. 5:**
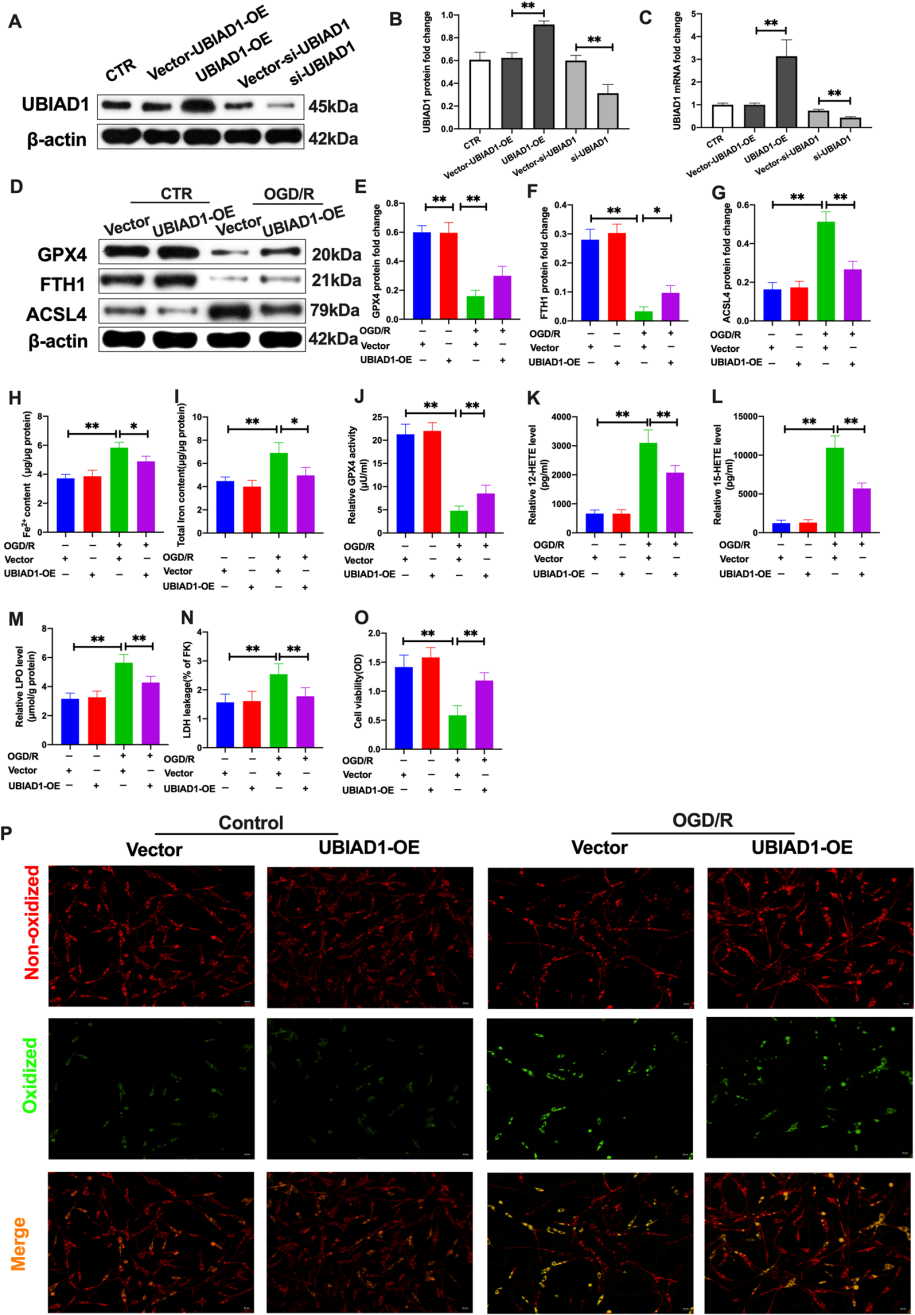
Overexpression of UBIAD1 improved OGD/R-induced ferroptotic neuronal death and lipid peroxidation in vitro. **A**–**C** The protein and mRNA expression of UBIAD1 in neurons as quantified with western blotting and PCR assay after overexpression and knockdown of UBIAD1 expression in neurons. **D**–**G** The protein expression of GPX4, FTH1, and ASCL4 in neurons as revealed by western blotting assay. **H**, **I** Content of Fe^2+^ and total iron in neurons as evaluated with the iron assay kit. **J** GPX4 activity in neurons as determined using GPX4 activity assay kit. **K**, **L** 12-HETE and 15-HETE levels in neurons as detected with ELISA assay kits. **M** LPO level in neurons as measured with the lipid peroxidation assay kit. **N** LDH level in neurons as determined with the LDH assay kit. **O** Results of MTT assay showing cell viability of neurons. **P** Lipid peroxidation in neurons based on BODIPY 581/591 C11 staining (Scale bar = 50 μm). n = 3, all data are expressed as the mean ± SD, *P < 0.05, **P < 0.01; CTR + vector-UBIAD-OE group vs. OGD/R + vector-UBIAD-OE group and OGD/R + vector-UBIAD-OE group vs. OGD/R + UBIAD-OE group

The roles of UBIAD1 in ferroptotic neuronal death and lipid peroxidation following cerebral I/R insult were first evaluated in vitro. UBIAD1 was overexpressed and knocked down in primary neurons through lentiviral and siRNA transfection, respectively. Western-blot and PCR analyses revealed that overexpressions of UBIAD1 (UBIAD1-OE group) significantly upregulated mRNA and protein levels in neurons, compared to the corresponding vector group (vector-UBIAD1-OE) (Fig. 5A–C). In the UBIAD1-siRNA group (UBIAD1-siRNA), there were marked reductions in mRNA and protein levels, relative to the corresponding vector group (vector-UBIAD1-siRNA) (Fig. 5A–C). Thereafter, we evaluated the effects of UBIAD1-overexpression on ferroptosis and lipid peroxidation under OGD/R-induced insult in primary neurons. Western-blot revealed that inhibition of GPX4 and FTH1 protein levels was improved in the UBIAD1-overexpression group under OGD/R conditions, compared to the vector-UBIAD1-OE group (Fig. 5D–F). Meanwhile, overexpressions of UBIAD1 in neurons significantly suppressed ASCL4 protein levels after OGD/R insults, relative to vector-UBIAD1-OE group (Fig. 5D, G). Furthermore, compared to vector-UBIAD1-OE group, UBIAD1 overexpression markedly inhibited OGD/R damage-mediated increase in Fe^2+^ and total iron levels (Fig. 5H, I). Besides, lipid peroxidation levels were markedly reduced in the UBIAD1-overexpression group, including upregulation of GPX4 activities as well as downregulation of 12-HETE and 15-HETE levels, relative to the vector-UBIAD1-OE group (Fig. 5J–L). After OGD/R insult, elevated levels of LPO and LDH levels were suppressed in UBIAD1 overexpression neurons, compared to the vector-UBIAD1-OE group (Fig. 5M, N). Meanwhile, UBIAD1 overexpression markedly rescued the decreased cell viability of damaged neurons in OGD/R, relative to the vector-UBIAD1-OE group (Fig. 5O). In addition, green oxidized fluorescence in UBIAD1 overexpression neurons was decreased after OGD/R insult, compared to the vector-UBIAD1-OE group (Fig. 5P). These findings imply that UBIAD1 overexpression reversed OGD/R-induced ferroptotic neuronal death and lipid peroxidation.

**Fig. 6:**
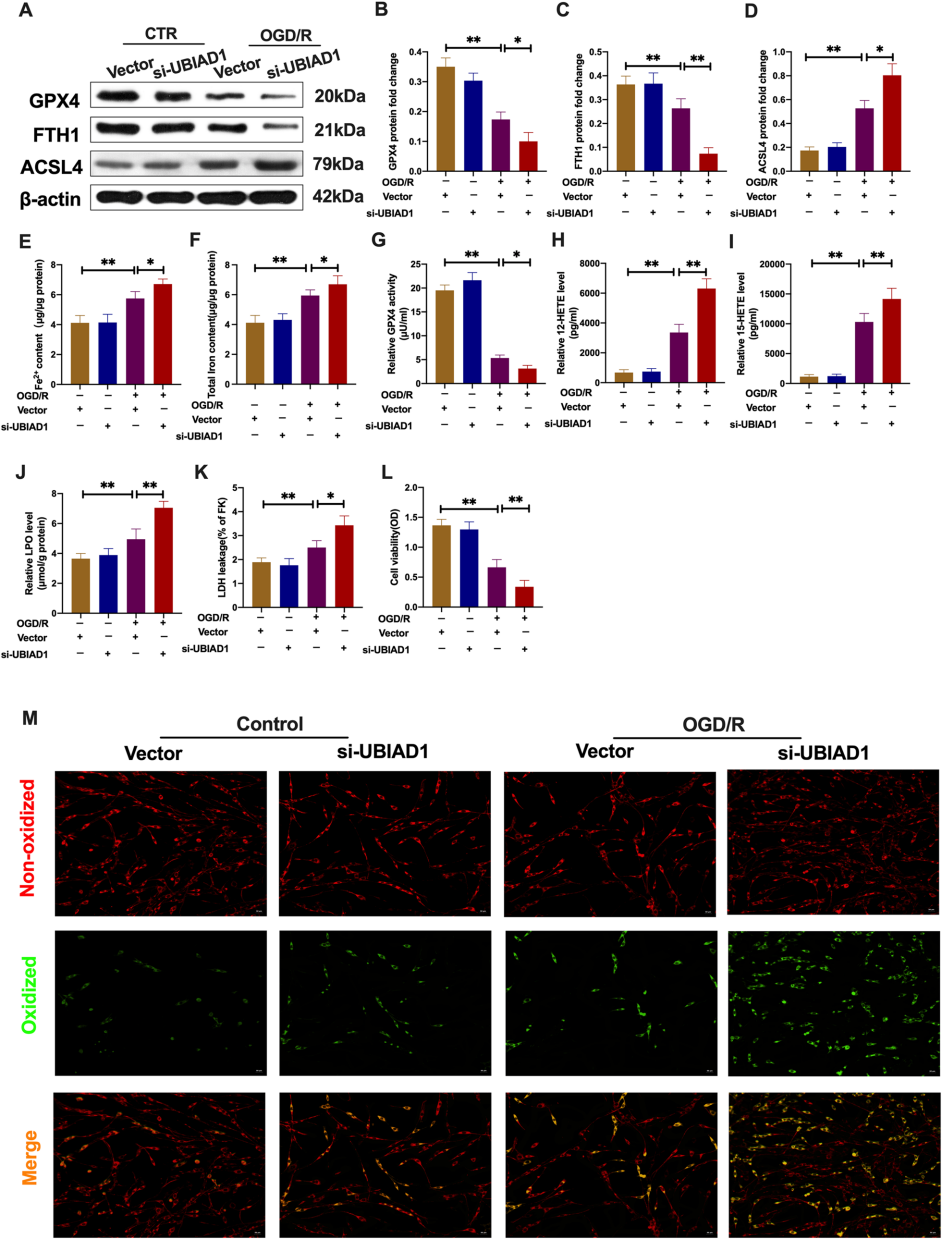
UBIAD1 knockdown aggravated OGD/R-induced ferroptotic neuronal death and lipid peroxidation in vitro. **A**–**D** The protein expression of GPX4, FTH1, and ASCL4 in neurons as determined by western blotting. **E**, **F** Content of Fe^2+^ and total iron in neurons as evaluated with the iron assay kit. **G** GPX4 activity in neurons as assayed using the GPX4 activity assay kit. **H**, **I** 12-HETE and 15-HETE levels in neurons as detected by ELISA assay kits. **J** LPO level in neurons as quantified with lipid peroxidation assay kit. **K** LDH level in neurons as determined using LDH assay kit. **L** Results of MTT assay showing viability of neurons. **M** Lipid peroxidation in neurons based on BODIPY 581/591 C11 staining (Scale bar = 50 μm). n = 3, all data are expressed as the mean ± SD, *P < 0.05, **P < 0.01; CTR + vector-UBIAD-siRNA group vs. OGD/R + vector-UBIAD-siRNA group and OGD/R + vector-UBIAD-siRNA group vs. OGD/R + UBIAD-siRNA group

Since upregulation of UBIAD1 blocked ferroptosis and lipid peroxidation in damaged neurons upon OGD/R, we evaluated the effects of UBIAD1 knockdown in neurons (UBIAD1-siRNA group). As shown in Fig. 6A–C, OGD/R downregulated GPX4 and FTH1 protein levels in UBIAD1 knock-down group, compared to the vector-UBIAD1-siRNA group. Besides, ACSL4 protein levels were increased in the suppressed UBIAD1 group, relative to the vector-UBIAD1-siRNA group (Fig. 6D). Elevated Fe^2+^ and total iron levels in impaired neurons after OGD/R led to acerbation in the UBIAD1-siRNA group, compared to the vector-UBIAD1-siRNA group (Fig. 6E, F). In tandem with the above results, GPX4 activities were markedly suppressed while 12-HETE, 15-HETE, LPO and LDH levels were significantly increased in neurons of the UBIAD1 knock-down group, relative to the vector-UBIAD1-siRNA group of OGD/R (Fig. 6G–K). Further, cell viabilities were markedly downregulated while green oxidized fluorescence was upregulated in insulted neurons of the UBIAD1-siRNA group, relative to the vector-UBIAD1-siRNA group (Fig. 6L, M).

In summary, UBIAD1 is a preferred modulator for mitigating OGD/R-induced ferroptotic neuronal death. Thus, subsequent assays evaluated whether UBIAD1 overexpression could improve brain tissue damage by alleviating I/R-mediated lipid peroxidation and ferroptosis in the brain.

### UBIAD1 overexpression improved brain tissue impairment induced by I/R-mediated lipid peroxidation and ferroptosis in vivo

**Fig. 7:**
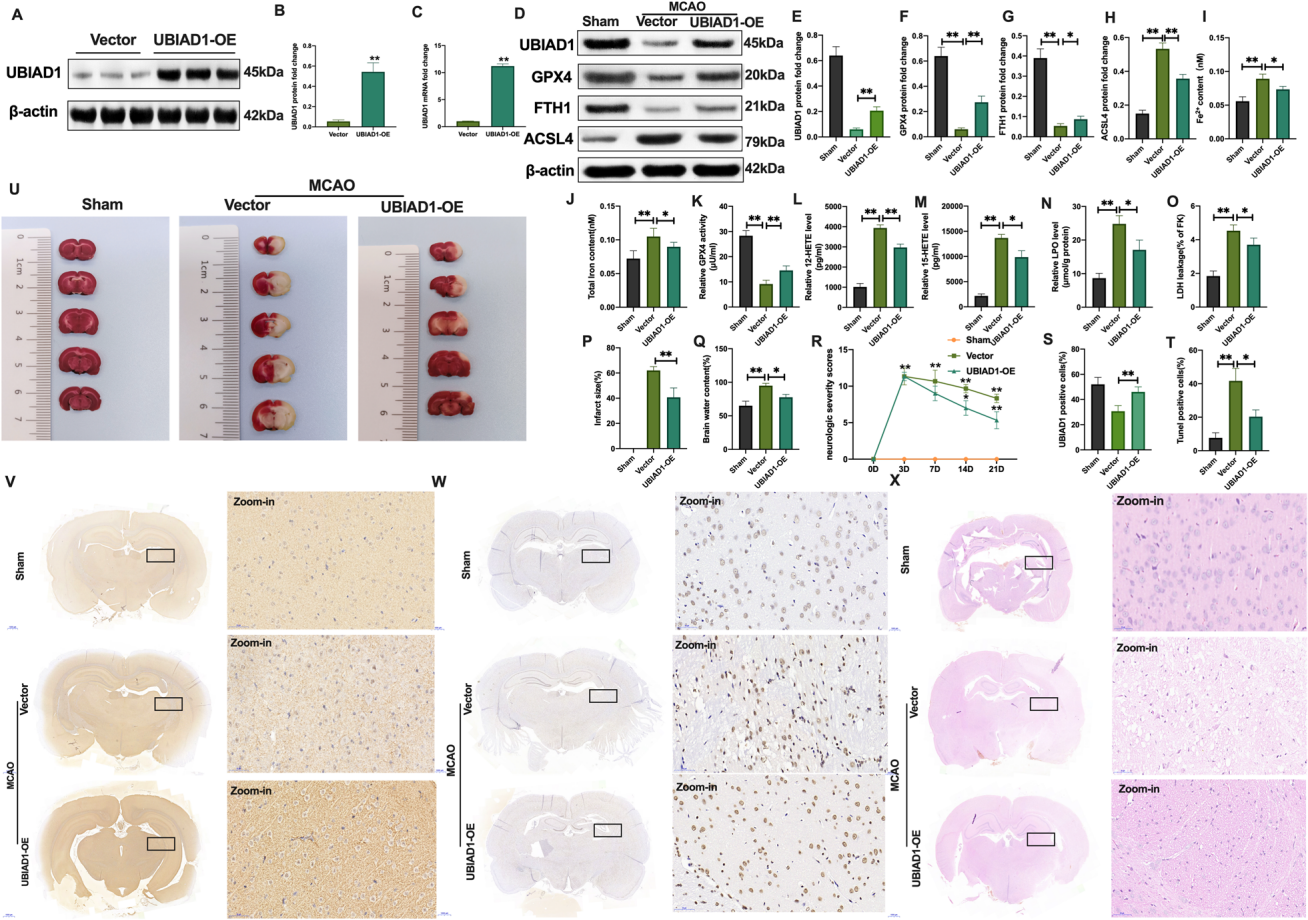
Overexpression of UBIAD1 suppressed brain injury induced by I/R-mediated lipid peroxidation and ferroptosis in vivo. **A**–**C** Overexpression of UBIAD1 by adeno-associated virus transfection as confirmed by western blotting and PCR assay in rats brain tissues. **D**–**H** The protein expression of UBIAD1, GPX4, FTH1, and ASCL4 in rat brain tissues after MCAO as determined by western blotting. **I**, **J** Content of Fe^2+^ and total iron in rat brain tissues after MCAO as evaluated using iron assay kit. **K** GPX4 activity in rat brain tissues after MCAO as assayed with the GPX4 activity assay kit. **L**, **M** 12-HETE and 15-HETE levels in rat brain tissues after MCAO as detected by ELISA assay kits. **N** LPO level in rat brain tissues after MCAO as determined with lipid peroxidation assay kit. **O** LDH level in rat brain tissues after MCAO determined by LDH assay kit. **P** and **U** Infarct size in whole rat brain tissues after MCAO as revealed by TTC staining. **Q** Brain water content after MCAO. **R** Confirmation of neurological severity scores after MCAO. **S** and **V** Protein level of UBIAD1 in rat brain tissues after MCAO as examined using immunohistochemical staining (Scale bar = 50 μm). **T** and **W** Neuronal death in rat brain tissues after MCAO as determined by the TUNEL staining assay (Scale bar = 50 μm). **X** HE staining in rat brain tissues after MCAO (Scale bar = 50 μm). n = 6 rats/group, all data are expressed as the mean ± SD, *P < 0.05, **P < 0.01; sham group vs. MCAO + vector-UBIAD1-AAV group and MCAO + vector-UBIAD1-AAV group vs. MCAO + UBIAD1-AAV group

Elevated UBIAD1 levels were achieved by transfection of an adeno-associated virus (AAV) into rat cortex and ipsilateral striatum posterior [35]. There were notable improvements in UBIAD1 mRNA and protein levels in rat brains following injection with AAV encoding UBIAD1 (Fig. 7A–C). After MCAO insult, downregulated protein levels and positive UBIAD1 cells were enhanced in the UBIAD-AAV + MCAO group, compared to the empty-UBIAD1-AAV + MCAO group (Fig. 7D, E, S, V). Furthermore, after MCAO operation, positive GPX4 and FTH1 protein levels were increased in UBIAD1-AAV infected rat brain tissues, compared to the empty-UBIAD1-AAV + MCAO group (Fig. 7D, F, G). Besides, UBIAD1 overexpressions significantly inhibited the enhanced expressions of ACSL4 protein levels in the UBIAD-AAV + MCAO group, compared to the empty-UBIAD1-AAV + MCAO group (Fig. 7D, H). The AAV-UBIAD1 treated MCAO rats exhibited suppressed Fe^2+^ and total iron levels, compared to the empty-UBIAD1-AAV + MCAO treated group (Fig. 7I, J). Lipid peroxidation, enhanced GPX4 activities, markedly declined 12-HETE, 15-HETE production, as well as significantly reduced LPO and LDH levels were noted in the UBIAD1-AAV infected MCAO group, compared to the empty-UBIAD1-AAV + MCAO group (Fig. 7K–O).

Subsequently, we determined whether overexpressed UBIAD1 could rescue brain impairments after MCAO operation. The TTC staining revealed that the infarct size of brain tissues was significantly suppressed in AAV-UBIAD1 transfected MCAO group, compared to the vector-UBIAD1-AAV + MCAO group (Fig. 7P, U). This phenomenon was accompanied by markedly inhibited brain water levels in the AAV-UBIAD1 treated group following MCAO, compared to the vector-UBIAD1-AAV + MCAO group (Fig. 7Q). In addition, the UBIAD1-AAV infected group exhibited markedly low neurologic severity scores on the 7th, 14th and 21st days following MCAO operation, compared to the vector-UBIAD1-AAV + MCAO group (Fig. 7R). Besides, there was a significant reduction in percentage of TUNEL positive cells in UBIAD1-AAV treated group, relative to the vector-UBIAD1-AAV + MCAO group (Fig. 7T, W). Similarly, HE staining revealed markedly improved brain tissue injury after UBIAD1-AAV transfection in MCAO rats, compared to the empty-UBIAD1-AAV + MCAO group (Fig. 7X).

These findings confirm that UBIAD1 overexpression mitigates brain tissue insult caused by I/R-mediated lipid peroxidation and ferroptosis. Thus, we analyzed the potential neuroprotective mechanisms of UBIAD1 on cerebral I/R-induced ferroptotic neuronal death and lipid peroxidation.

### Elevated UBIAD1 expression inhibited oxidative stress by alleviating mitochondrial dysfunction in OGD/R-mediated ferroptosis in vitro

**Fig. 8:**
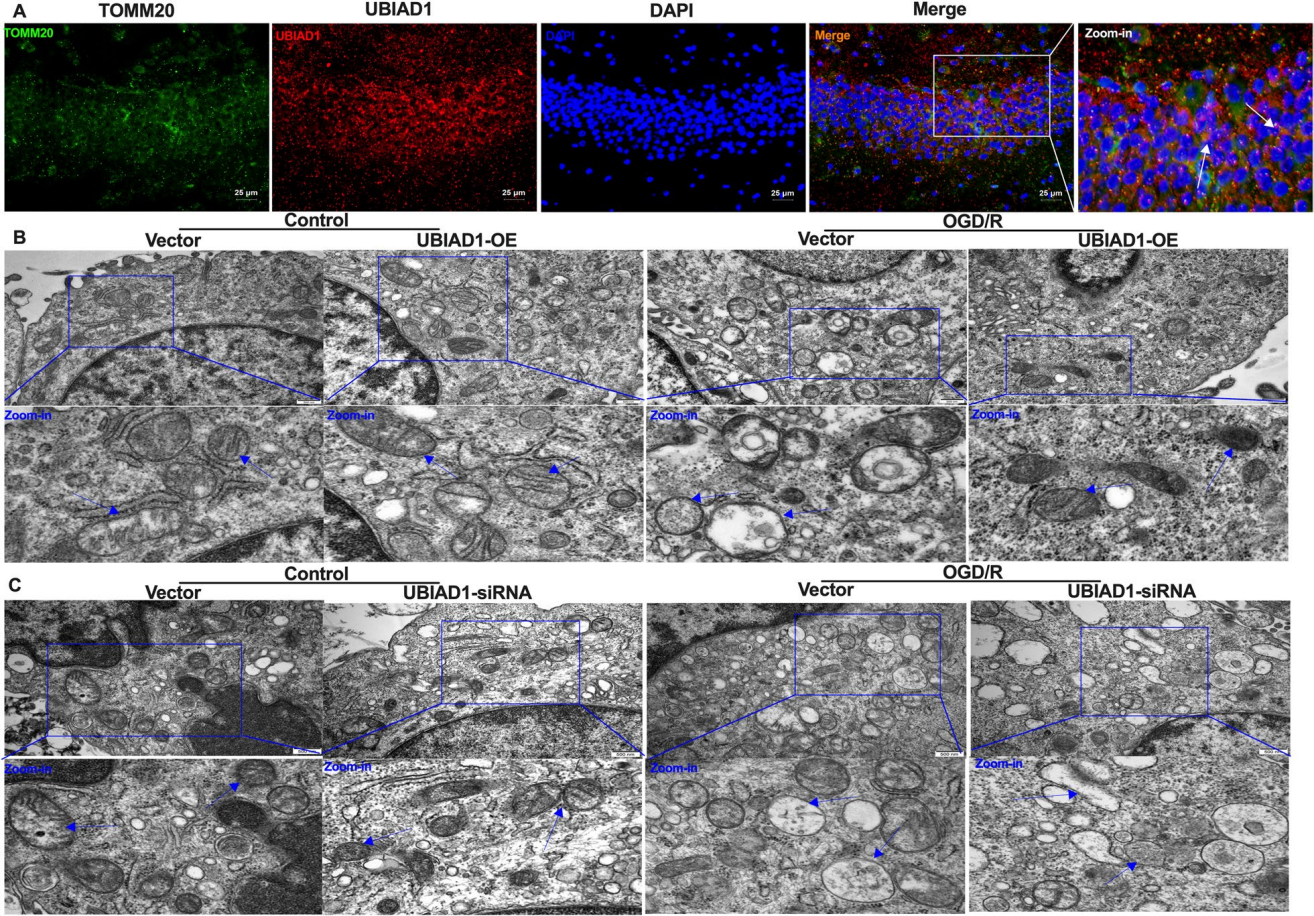
Overexpression of UBIAD1 restored mitochondrial fragmentation in OGD/R-mediated ferroptosis in vitro. **A** Confocal images showing co-localization of UBIAD1 (red) with mitochondria marker TOMM20 (green) (Scale bar = 25 μm). **B** Changes to the mitochondrial morphology and ultrastructure in UBIAD1-overexpressing neurons after OGD/R insult as examined with TEM (Scale bar = 500 nm). **C** Changes in mitochondrial morphology and ultrastructure in UBIAD1-siRNA neurons after OGD/R insult as revealed by TEM (Scale bar = 500 nm). n = 3, all data are expressed as the mean ± SD, *P < 0.05, **P < 0.01

Oxidative stress damage is a core molecular mechanism of ferroptosis in various I/R events. Mitochondrial dysfunction is a “hallmark” of oxidative programmed neuronal death upon cerebral I/R challenge [36]. Thus, we investigated whether UBIAD1 can protect against OGD/R insult-induced mitochondrial dysfunction in ferroptotic neuronal death. Furthermore, immunofluorescence staining was performed to illustrate subcellular organelle localization of UBIAD1, while TOMM20, a typical mitochondrial marker, was used to study mitochondrial localization [37]. It was found that TOMM20 was co-localized with UBIAD1 in the hippocampus region of rat brain tissues, indicating that UBIAD1 is localized in the mitochondria (Fig. 8A). Thereafter, we investigated the roles of UBIAD1 in mitochondrial morphology and functions under OGD/R conditions. We used TEM to analyze alterations in mitochondrial ultrastructures in damaged neurons after OGD/R insult. To assess changes in mitochondrial morphologies, mitochondrial shapes were analyzed using the grades criteria [35]: Class I: more than four cristae, Class II: two to three cristae, Class III: less than one cristae; Class A: dense matrix in the mitochondria, Class B: hypodense matrix in swollen mitochondria (Additional file 1: A). Under non-OGD/R conditions, the vector-UBIAD1-OE group showed about 82% Class I and 88% Class A neuronal mitochondria. Consistent with the non-OGD/R-vector-UBIAD1-OE group, the UBIAD1-OE group showed about 87% Class I and 91% Class A neuronal mitochondria upon non-OGD/R (Fig. 8B, Additional file 1: B, C). In the OGD/R-insulted group, the vector-UBIAD1-OE neuronal mitochondria subjected to severe insult were approximately 78% Class III and 92% Class B, accompanied by a marked decrease in mitochondrial length compared to the non-OGD/R + vector-UBIAD1-OE group (Fig. 8B, Additional file 1: B–D). The damaged mitochondrial shapes were obviously restored by approximately 30% Class III and 54% Class B. Mitochondrial length were significantly longer in the OGD/R + UBIAD1-OE group, compared to the OGD/R + vector-UBIAD1-OE group (Fig. 8B, Additional file 1: B–D). Thus, overexpressed UBIAD1 effectively alleviated OGD/R-induced impairments of mitochondrial fragmentation.

**Fig. 9:**
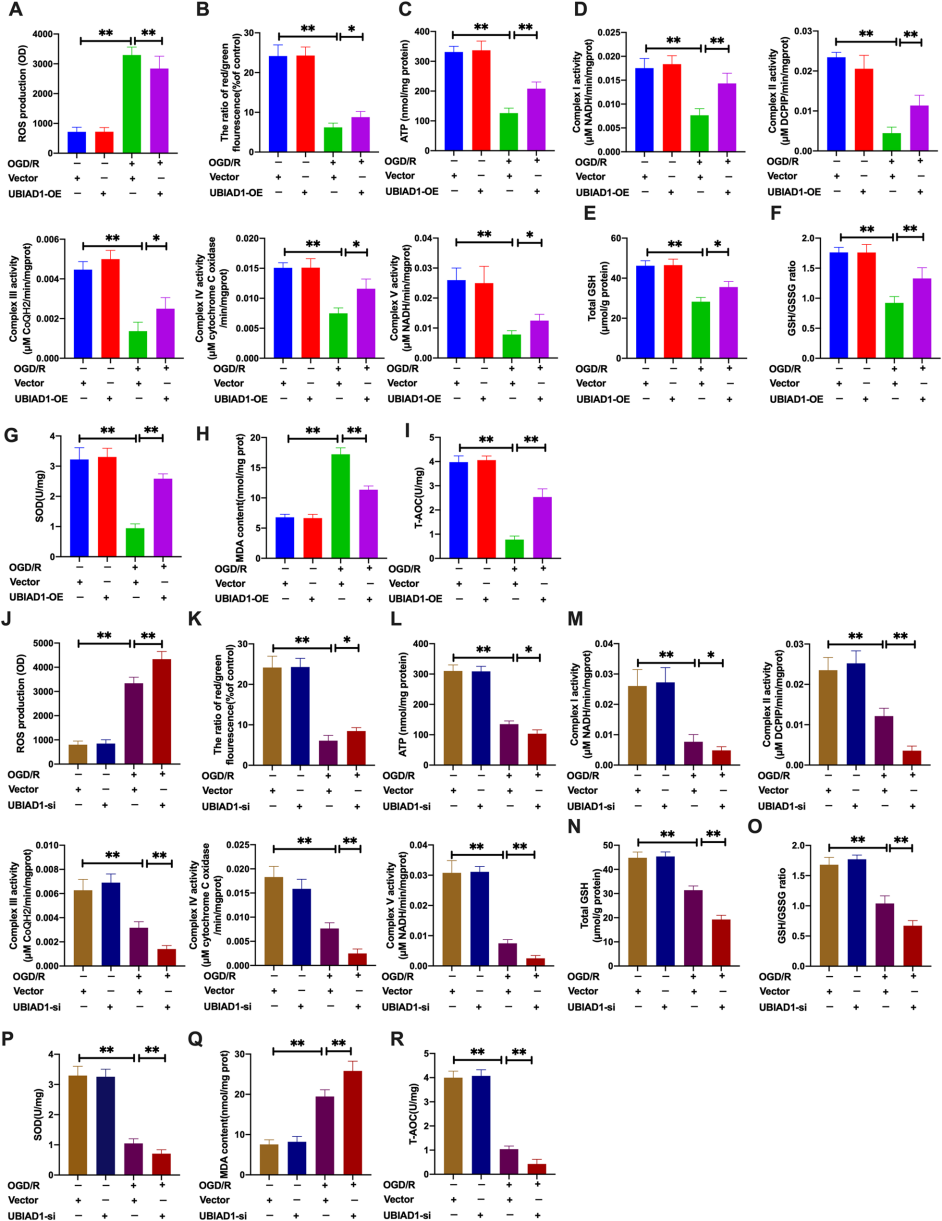
Overexpression of UBIAD1 inhibited oxidative stress by restoring mitochondrial dysfunction in OGD/R-mediated ferroptosis in vitro. The levels of oxidative stress in UBIAD1-OE and UBIAD1-siRNA neurons, and corresponding experimental groups. **A** and **J** ROS production as detected using DCFH-SA assay. **B** and **K** Level of mitochondrial membrane potential in various experimental groups as determined using JC-1 staining. **C** and **L** ATP production in various experimental groups as evaluated with the ATP assay kit. **D** and **M** The activity of mitochondrial complexes I, II, III, IV, and V in various experimental groups. **E**, **F** and **N**, **O** The level of total GSH and GSH/GSSG ratio in various experimental groups as determined by GSH assay kit. **G** and **P** Changes in SOD production in various experimental groups as detected with the WST-8 assay kit. **H** and **Q** The level of MAD generation in various experimental groups as determined with the lipid peroxidation assay kit. **I** and **R** The level of T-AOC in various experimental groups as evaluated using ABTS assay kit. N = 3, all data are expressed as the mean ± SD, *P < 0.05, **P < 0.01; CTR + vector-UBIAD-OE group vs. OGD/R + vector-UBIAD-OE group and OGD/R + vector-UBIAD-OE group vs. OGD/R + UBIAD-OE group; CTR + vector-UBIAD-siRNA group vs. OGD/R + vector-UBIAD-siRNA group and OGD/R + vector-UBIAD-siRNA group vs. OGD/R + UBIAD-siRNA group

Next, we assessed the effects of UBIAD1-overexpression on mitochondrial functions as well as on the levels of oxidative stress upon OGD/R condition [18]. Compared to the OGD/R + vector-UBIAD1-OE group, overexpressed UBIAD1 in neurons inhibited ROS levels and enhanced mitochondrial membrane potential (ΔΨm) as well as ATP production upon OGD/R insult (Fig. 9A–C and Additional file 2: A, B). Besides, we assessed the effects of elevated UBIAD1 levels on mitochondrial metabolism using five protein complexes in the mitochondrial electron transport chain (ETC) [38]. Upregulated UBIAD1 significantly increased the activities of mitochondrial complexes in the OGD/R + UBIAD1-OE group, compared to the OGD/R + vector-UBIAD1-OE group (Fig. 9D). Then, we determined oxidative stress levels in insulted neurons. As shown in Fig. 9E, F, UBIAD1 overexpression significantly increased total GSH and GSH/GSSG levels in OGD/R insults, compared to OGD/R + vector-UBIAD1-OE group. Moreover, overexpressed UBIAD1 in the OGD/R + UBIAD1-OE group increased SOD and T-AOC levels while suppressing MDA levels, compared to the OGD/R + vector-UBIAD1-OE group (Fig. 9G–I). Thus upregulated UBIAD1 levels inhibited oxidative stress-associated damage by reversing OGD/R insult-mediated mitochondrial dysfunctions in neurons.

To confirm these findings, we transfected UBIAD1 siRNA into neurons (Fig. 5). The non-OGD/R-vector-UBIAD1-siRNA group had about 90% Class I and 90% Class A neuronal mitochondria (Fig. 8C, Additional file 1: E, F). Consistent with the non-OGD/R-vector-UBIAD1-siRNA group, the non-OGD/R-UBIAD1-siRNA group showed approximately 88% Class I and 86% Class A neuronal mitochondria (Fig. 8C, Additional file 1: E, F). Meanwhile, there was 75% Class III and 87% Class B impaired mitochondria in the OGD/R + vector-UBIAD1-siRNA group (Fig. 8C, Additional file 1: E, F). The OGD/R + UBIAD1-siRNA group showed more severe injured mitochondria (about 90% Class III and 96% Class B) as well as significantly shorter mitochondrial length compared to the OGD/R + vector-UBIAD1-siRNA group (Fig. 8C, Additional file 1: E–G). Therefore, suppression of UBIAD1 aggravated mitochondrial morphology and ultrastructural damage during OGD/R challenge in neurons.

Moreover, compared to the OGD/R + vector-UBIAD1-siRNA group, UBIAD1-siRNA neurons significantly increased ROS generation and downregulated mitochondrial membrane potential (ΔΨm) as well as ATP production during OGD/R insult (Fig. 9J–L and Additional file 2: C, D). Besides, the five mitochondrial complexes were significantly decreased in the OGD/R + UBIAD1-siRNA group relative to the OGD/R + vector-UBIAD1-siRNA group (Fig. 9M). Altered antioxidant capacities were noted in UBIAD1 knock-down neurons. The obviously downregulation of total GSH, GSH/GSSG, and SOD production as well as T-AOC levels were observed in the OGD/R + UBIAD1-siRNA grou compared to the OGD/R + vector-UBIAD1-siRNA group (Fig. 9N–P, R). On the contrary, compared to the OGD/R + vector-UBIAD1-siRNA group, MDA levels were markedly elevated in the OGD/R + UBIAD1-siRNA group (Fig. 9Q).

In conclusion overexpressed UBIAD1 exerts protective effects by enhancing antioxidant capacities via restoring impaired mitochondrial ultrastructures, morphology and functions in OGD/R insulted neurons. Then, we determined whether UBIAD1 inhibits ferroptotic neuronal death and lipid peroxidation by suppressing oxidative stress via attenuation of mitochondrial dysfunction in cerebral I/R challenge.

### Elevated UBIAD1 levels inhibited ferroptotic neuronal death and lipid peroxidation by suppressing oxidative stress via attenuation of OGD/R injury-induced mitochondrial impairment in vitro

**Fig. 10:**
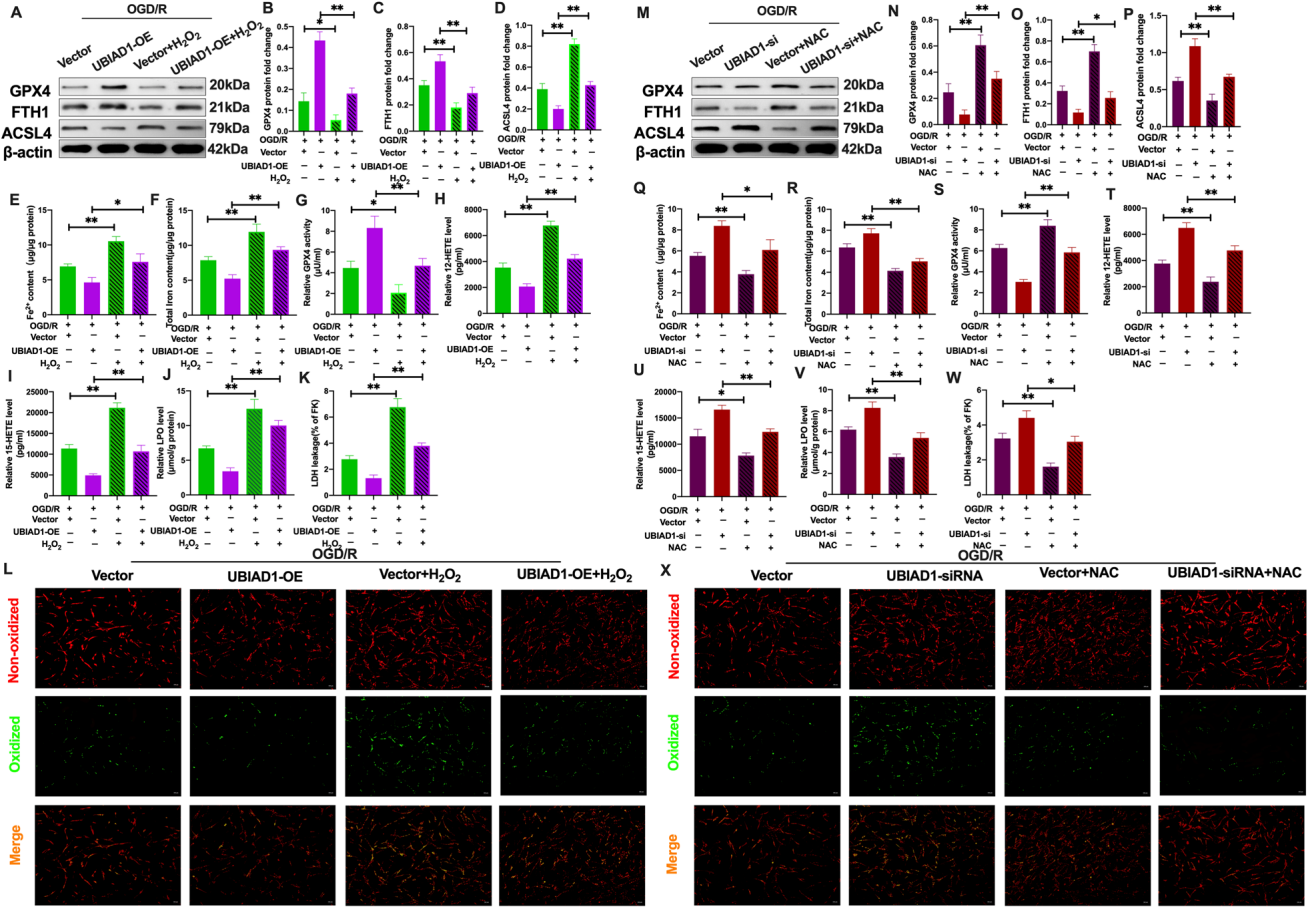
Overexpression of UBIAD1 inhibited ferroptotic neuronal death and lipid peroxidation by restraining OGD/R-induced oxidative stress in vitro. H_2_O_2_ and NAC were pretreated with UBIAD1-OE and UBIAD1-siRNA neurons before OGD/R insult. **A**–**D** and **M**–**P** The protein expression of GPX4, FTH1, and ASCL4 as determined by western blotting. **E**, **F** and **Q**, **R** Content of Fe^2+^ and total iron in various experimental groups as evaluated using iron assay kit. **G** and **S** GPX4 level in various experimental groups. **H**, **I** and **T**, **U** 12-HETE and 15-HETE levels in various experimental groups as detected with ELISA assay kits. **J** and **V** The level of LPO in the various experimental groups as assayed using lipid peroxidation assay kit. **K** and **W** The level of LDH in various experimental groups as evaluated with the LDH assay kit. **L** and **X** Lipid peroxidation in neurons based on BODIPY 581/591 C11 staining (Scale bar = 50 μm). n = 3, all data are expressed as the mean ± SD, *P < 0.05, **P < 0.01; OGD/R + vector -UBIAD1-OE group vs. OGD/R + vector -UBIAD1-OE + H_2_O_2_ group, OGD/R + UBIAD1-OE group vs. OGD/R + UBIAD1-OE + H_2_O_2_ group; OGD/R + vector -UBIAD1-siRNA group vs. OGD/R + vector-UBIAD1-siRNA + NAC group, OGD/R + UBIAD1-siRNA group vs. OGD/R + UBIAD1-siRNA + NAC group

The mitochondrial oxidative stress-inducing agent (H_2_O_2_) was applied to UBIAD1-overexpression neurons to modulate the antioxidant capacities [39]. Then, oxidative stress levels were determined in UBIAD1-overexpression neurons. Compared to the OGD/R + UBIAD1-OE group, SOD and T-AOC levels were significantly suppressed following H_2_O_2_ treatment in the OGD/R + UBIAD1-OE + H_2_O_2_ group (Additional file 3: A and C). Besides, MDA levels were obviously suppressed in the OGD/R + UBIAD1-OE group while being significantly enhanced in the OGD/R + UBIAD1-OE + H_2_O_2_ group following H_2_O_2_ treatment (Additional file 3: B). These findings imply that H_2_O_2_ suppressed antioxidant capacities in UBIAD1 overexpression neurons. Next, we assessed the relationship between UBIAD1-mediated mitochondrial oxidative stress and ferroptosis upon OGD/R insult. As shown in Fig. 10A–C, GPX4 and FTH1 protein levels were markedly inhibited after H_2_O_2_ exposures in the OGD/R + UBIAD1-OE + H_2_O_2_ group, compared to the OGD/R + UBIAD1-OE group (Fig. 10A–C). Further, relative to the OGD/R + UBIAD1-OE group, the suppressed ASCL4 protein levels were elevated after combination with H_2_O_2_ intervention in the OGD/R + UBIAD1-OE + H_2_O_2_ group (Fig. 10A, D). Compared to the OGD/R + UBIAD1-OE group, the intervention OGD/R + UBIAD1-OE + H_2_O_2_ neurons group exhibited higher Fe^2+^ and total iron levels (Fig. 10E, F). Further, there was significant exacerbation of lipid peroxidation indices in the OGD/R + UBIAD1-OE-H_2_O_2_ group, including suppressed activities of GPX4, enhanced 12-HETE and 15-HETE levels, as well as LPO, LDH and increased green oxidized fluorescence following H_2_O_2_ in the OGD/R + UBIAD1-OE-H_2_O_2_ group, relative to the OGD/R + UBIAD1-OE group (Fig. 10G–L). These results indicate that enhanced mitochondrial-mediated oxidative stress in UBIAD1-overexpression neurons aggravates OGD/R insult-induced ferroptotic neuronal death.

To confirm the role of UBIAD1-regulated mitochondrial dysfunction in ferroptosis, the mitochondrial oxidative stress-antioxidative mediator, NAC, was used in UBIAD1 knockdown neurons following OGD/R challenge [40]. There was a marked increase in antioxidative capacities in UBIAD1-siRNA neurons after NAC treatment. This was shown by elevated SOD as well as T-AOC levels and suppressed MDA levels in the OGD/R + UBIAD1-siRNA + NAC group, compared to the OGD/R + UBIAD1-siRNA group (Additional file 3: D–F). In addition, the downregulated GPX4 and FTH1 protein levels were markedly inhibited by combined NAC treatment in OGD/R + UBIAD1-siRNA + NAC group, relative to the OGD/R + UBIAD1-siRNA group (Fig. 10M–O). Furthermore, ASCL4 protein levels were suppressed in the OGD/R + UBIAD1-siRNA + NAC group, relative to the OGD/R + UBIAD1-siRNA group (Fig. 10M, P). Moreover, there were significantly suppressed Fe^2+^ and total iron levels following NAC treatment in OGD/R + UBIAD1-siRNA + NAC group, relative to the OGD/R + vector-UBIAD1-siRNA and OGD/R + UBIAD1-siRNA groups, respectively (Fig. 10Q, R). In the NAC intervention, levels of lipid peroxidation indices were significantly mitigated in the OGD/R + UBIAD1-siRNA + NAC group, including enhanced activities of GPX4, downregulated levels of 12-HETE and 15-HETE, as well as LPO, LDH and alleviated green oxidized fluorescence, compared to the OGD/R + UBIAD1-siRNA group (Fig. 10S–X).

These findings imply that UBIAD1 exerted neuroprotective effects on cerebral I/R-induced ferroptotic neuronal death and lipid peroxidation by improving antioxidative capacities via attenuating mitochondrial impairment in insulted neurons.

### Overexpressed UBIAD1 elevated antioxidative capacities by reversing Golgi apparatus dysfunctions in OGD/R-regulated ferroptosis in vitro

**Fig. 11:**
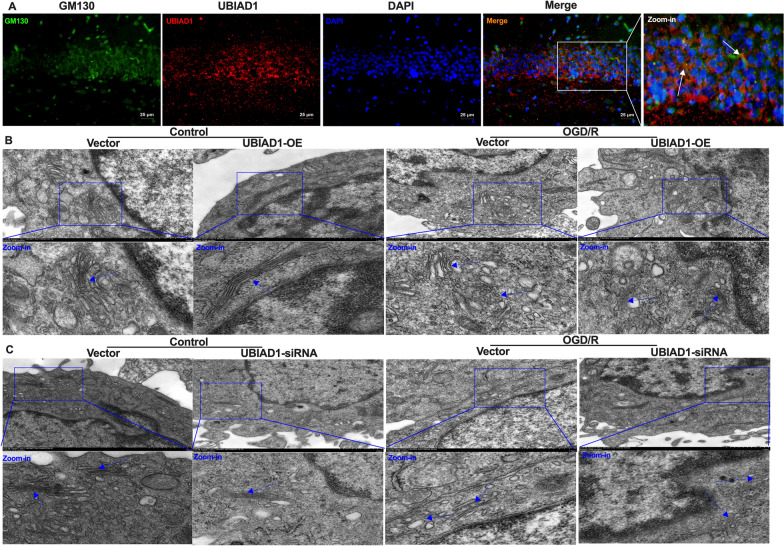
Overexpression of UBIAD1 elevated antioxidative capacity by preserving Golgi apparatus fragmentation in OGD/R-induced ferroptosis in vitro. **A** Confocal images showing co-localization of UBIAD1 (red) with Golgi apparatus marker GM130 (green) (Scale bar = 25 μm). **B** Changes in the Golgi apparatus morphology and ultrastructure in UBIAD1-overexpressing neurons after OGD/R insult as observed by TEM (Scale bar = 500 nm). **C** Changes in Golgi apparatus morphology and ultrastructure in UBIAD1-siRNA neurons after OGD/R insult as examined under TEM (Scale bar = 500 nm). n = 3, all data are expressed as the mean ± SD, *P < 0.05, **P < 0.01

To elucidate on the potential mechanisms of UBIAD in ferroptosis after cerebral I/R damage, we investigated the effects of UBIAD1 on Golgi apparatus dysfunction in cerebral I/R. Figure 11A shows that GM130 was the most common morphological marker of the Golgi apparatus [41]. Merged figures revealed co-localization of green GM130 and red UBIAD1 fluorescence in rat brain tissues, indicating that UBIAD1 was also localized in the Golgi apparatus. Next, we investigated the effects of UBIAD1 on Golgi apparatus stress under OGD/R conditions. The Golgi apparatus ultrastructure was assessed using TEM and evaluated as previously reported [42]. The morphology of the Golgi apparatus is clearly illustrated in Additional file 4: A insets. As shown in Fig. 11B, C and Additional file 4: A, all primary neurons exhibited normal Golgi apparatus shapes and correct cis–trans polarity distribution. However, there were disruptions in Golgi apparatus polarity distribution that could not be recognized. Compared to the entire control group, Golgi apparatus structures in the neurons were converted into multiple mini-stacks after OGD/R insults (Fig. 11B, C). Moreover, after OGD/R insult, the longest length of Golgi cisternae was decreased in the OGD/R + vector-UBIAD1-OE group, compared to the CTR + vector-UBIAD1-OE group (Fig. 11B, Additional file 4: B). Consistent with this result, the shortest length of Golgi cisternae was shorter in the OGD/R + vector-UBIAD1-OE group than in the CTR + vector-UBIAD1-OE group (Fig. 11B, Additional file 4: C). However, compared to the OGD/R + vector-UBIAD1-OE group, the OGD/R + UBIAD1-OE group had a significantly increased in the longest length of Golgi cisternae (Fig. 11B, Additional file 4: B). There were no significant differences in the shortest length of the Golgi cisternae in OGD/R + UBIAD1-OE group and OGD/R + vector-UBIAD1-OE group (Fig. 11B, Additional file 4: C). Furthermore, OGD/R increased the width of Golgi apparatus or Golgi cisternae in the OGD/R + vecor-UBIAD1-OE group, relative to the CTR + vector-UBIAD1-OE group (Fig. 11B, Additional file 4: D, E). Moreover, compared to the OGD/R + vector-UBIAD1-OE group, the increase in the width of Golgi apparatus and Golgi cisternae were significantly inhibited in the OGD/R + UBIAD1-OE group (Fig. 11B, Additional file 4: D, E). These findings imply that UBIAD1 has a key role in maintaining the normal morphological structure of the Golgi apparatus.

To further investigate the effects of UBIAD1 on Golgi apparatus morphology, UBIAD1 was knocked down in the neurons after which changes in the Golgi apparatus were observed. Compared to the CTR + vector-UBIAD1-siRNA group, the OGD/R + vector-UBIAD1-siRNA group had a significantly decreased in the longest length of the Golgi cisternae (Fig. 11C, Additional file 4: F). However, there were no discernible differences in the shortest length of the Golgi cisternae between the CTR + vector-UBIAD1-siRNA group and the OGD/R + vector-UBIAD1-siRNA group (Fig. 11C, Additional file 4: G). Under OGD/R conditions, the OGD/R + UBIAD1-siRNA group exhibited a marked decrease in the longest length and shortest lengths of the Golgi cisternae, compared to the OGD/R + vector-UBIAD1-siRNA group (Fig. 11C, Additional file 4: F, G). Furthermore, the increase in the width of the Golgi apparatus was accompanied by an increase in the width of the Golgi cisternae in the OGD/R + vector-UBIAD1-siRNA group, compared to the CTR + vector-UBIAD1-siRNA group (Fig. 11C, Additional file 4: H, I). The larger width of the Golgi apparatus and the width of the Golgi cisternae were markedly enhanced in the OGD/R + UBIAD1-siRNA group, compared to the OGD/R + vector-UBIAD1-siRNA group (Fig. 11C, Additional file 4: H, I). These results suggest that upregulating UBIAD1 expressions protected the primary neurons from OGD/R injury-mediated disruptions of Golgi apparatus ultrastructure and morphology.

**Fig. 12:**
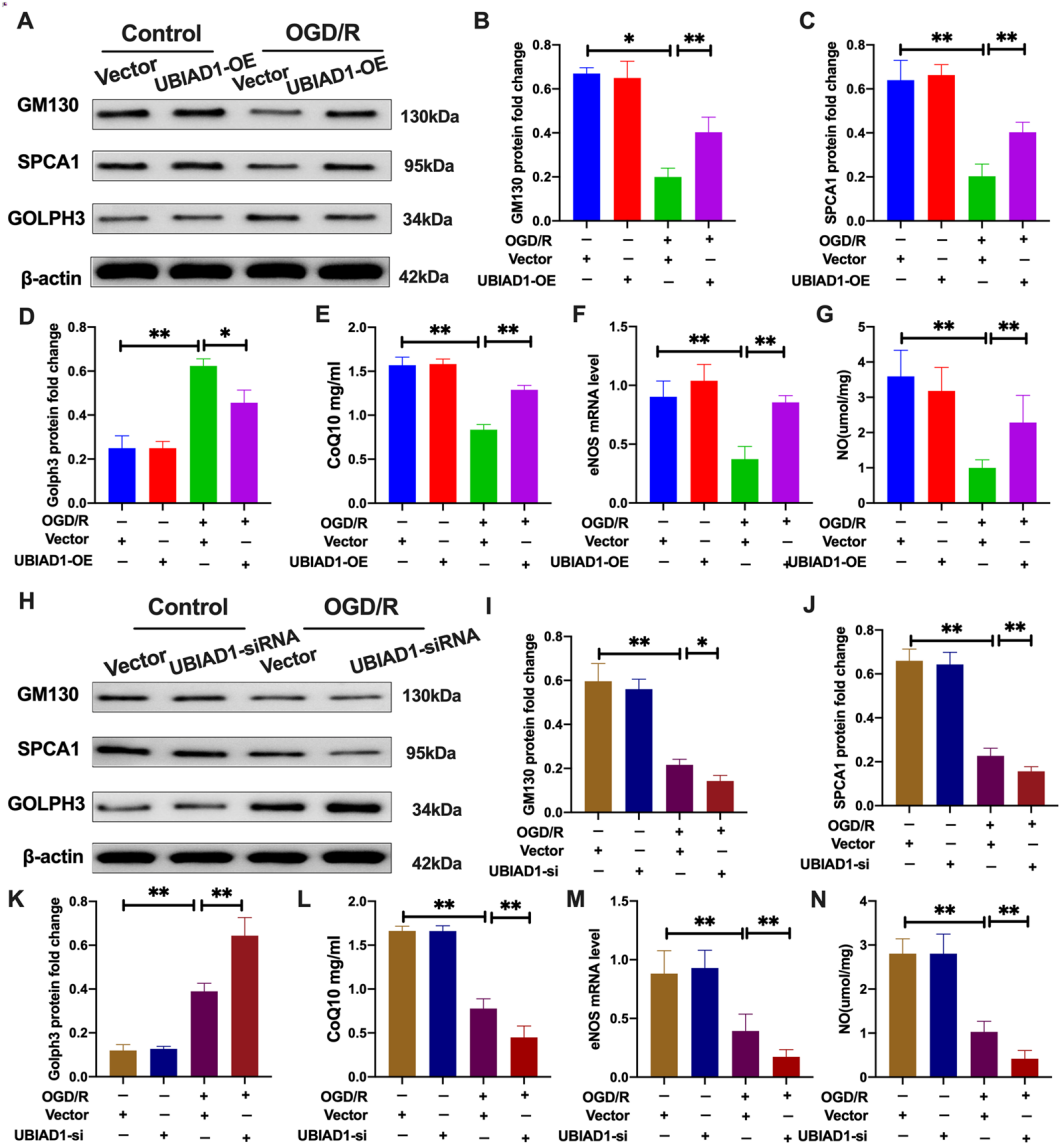
Upregulation of UBIAD1 mitigated Golgi apparatus stress associated with OGD/R-induced ferroptosis in vitro. **A**–**D** and **H**–**K** Protein expression of GM130, SPCA1, and GOLPH3 in various experimental groups as determined by western blotting. **E** and **L** Levels of CoQ10 production in various experimental groups as assayed using CoQ10 assay kit. **F** and **M** The mRNA expression of eNOS in neurons as detected using PCR assay. **G** and **N** Levels of NO in various experimental groups as assayed using NO assay kit. n = 3, all data are expressed as the mean ± SD, *P < 0.05, **P < 0.01; CTR + vector-UBIAD-OE group vs. OGD/R + vector-UBIAD-OE group and OGD/R + vector-UBIAD-OE group vs. OGD/R + UBIAD-OE group; CTR + vector-UBIAD-siRNA group vs. OGD/R + vector-UBIAD-siRNA group and OGD/R + vector-UBIAD-siRNA group vs. OGD/R + UBIAD-siRNA group

Then, the effects of OGD/R insult on Golgi apparatus functions were evaluated [41]. As shown in Fig. 12A–D, the OGD/R + UBIAD1-OE group exhibited significantly increased GM130 and SPCA1 protein levels [43] as well as markedly suppressed GOLPH3 protein levels [44], compared to the OGD/R + vector-UBIAD1-OE group. On the contrary, during OGD/R insult, the OGD/R + UBIAD1-siRNA group had markedly low GM130 as well as SPCA1 protein levels and markedly elevated GOLPH3 protein levels, relative to the OGD/R + vector-UBIAD1-siRNA group (Fig. 12H–K).

Biologically, UBIAD1 is involved in the biosynthesis of non-mitochondrial CoQ10 in the Golgi apparatus [21], which is directly associated with modulation of antioxidative capacities by regulating eNOS activities and NO signaling pathways [21]. As shown in Fig. 12E–G, the OGD/R + UBIAD1-OE group had significantly elevated CoQ10, eNOS and NO levels, compared to the OGD/R + vector + UBIAD1-OE group. However, the OGD/R + UBIAD1-siRNA group had remarkedly suppressed in the levels of CoQ10, eNOS and NO production, relative to the OGD/R + vector + UBIAD1-siRNA group (Fig. 12L–N).

Collectively, these findings imply that Golgi apparatus stress could have occurred in vitro after cerebral I/R insult, including the disruption of Golgi apparatus ultrastructure and morphology, as well as significant modification of Golgi apparatus-associated protein expressions. Furthermore, UBIAD1 overexpression alleviated Golgi apparatus stress, promoting antioxidative capacities by enhancing CoQ10 levels, elevating eNOS activities and eventually increasing NO levels in the neurons after OGD/R insult. Therefore, we determined whether UBIAD1-regulated ferroptosis and lipid peroxidation by elevating antioxidative capacities via preventing Golgi apparatus dysfunctions in injured neurons after OGD/R.

### Overexpressed UBIAD1 suppressed ferroptotic neuronal death and lipid peroxidation by elevating antioxidative capacities via mitigation of OGD/R insult-induced Golgi apparatus dysfunctions in vitro

**Fig. 13:**
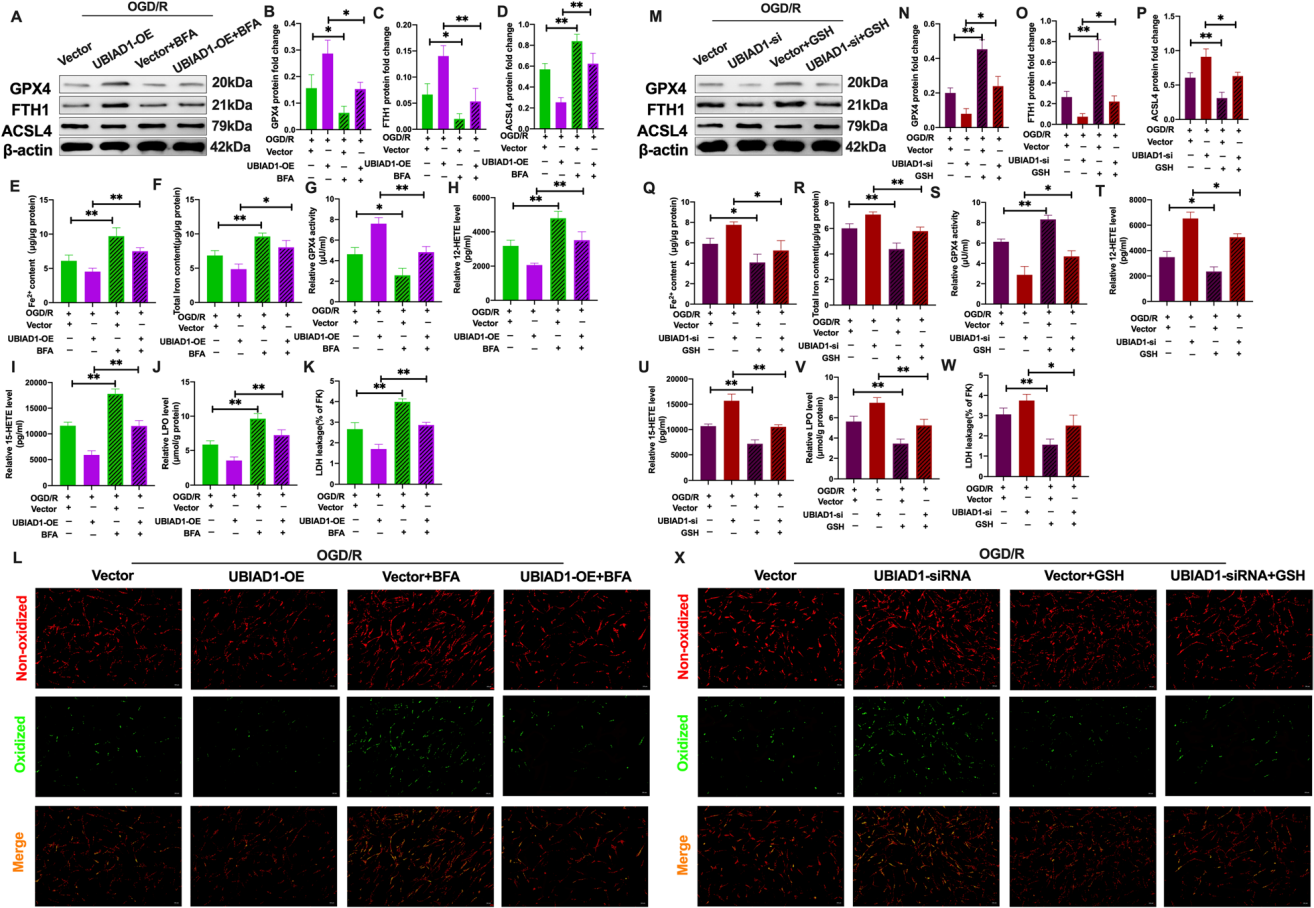
Overexpression of UBIAD1 reduced ferroptotic neuronal death by attenuating Golgi apparatus dysfunction caused by OGD/R insult in vitro. BFA and GSH were pretreated with UBIAD1-OE and UBIAD1-siRNA neurons in corresponding experimental groups before OGD/R insult. **A**–**D** and **M**–**P** The protein expression of GPX4, FTH1, and ASCL4 in various experimental groups as detected using western blotting. **E**, **F** and **Q**, **R** Content of Fe^2+^ and total iron in various experimental groups as evaluated using iron assay kit. **G** and **S** GPX4 activity in various experimental groups. **H**, **I** and **T**, **U** 12-HETE and 15-HETE levels in various experimental groups as determined using ELISA assay kits. **J** and **V** LPO level in various experimental groups. **K** and **W** The level of LDH in various experimental groups as evaluated using LDH assay kit. **L** and **X** Lipid peroxidation in neurons based on BODIPY 581/591 C11 staining (Scale bar = 50 μm). n = 3, all data are expressed as the mean ± SD, *P < 0.05, **P < 0.01; OGD/R + vector -UBIAD1-OE group vs. OGD/R + vector-UBIAD1-OE + BFA group, OGD/R + UBIAD1-OE group vs. OGD/R + UBIAD1-OE + BFA group; OGD/R + vector-UBIAD1-siRNA group vs. OGD/R + vector-UBIAD1-siRNA + GSH group, OGD/R + UBIAD1-siRNA group vs. OGD/R + UBIAD1-siRNA + GSH group

The Golgi apparatus stress-inducing agent (BFA) was used to overexpress UBIAD1 in neurons [19]. It was found that CoQ10 levels in OGD/R + UBIAD1-OE + BFA group were significantly suppressed, relative to the OGD/R + UBIAD-OE group (Additional file 5: A). Meanwhile, as shown in Fig. 13A–C, after BFA treatment, compared to the OGD/R + UBIAD1-OE group, GPX4 and FTH1 protein levels were significantly decreased in the OGD/R + UBIAD1-OE + BFA group. In contrast, negative expressions of the ASCL4 protein were markedly upregulated in the OGD/R + UBIAD1-OE + BFA group, relative to the OGD/R + UBIAD1-OE group (Fig. 13A, D). Furthermore, the suppressed Fe^2+^ and total iron levels in OGD/R + UBIAD1-OE neurons were markedly increased in the OGD/R + UBIAD1-OE + BFA group (Fig. 13E, F). Meanwhile, in the BFA intervention, the levels of lipid peroxidation indices were obviously exacerbated in the OGD/R + UBIAD1-OE + BFA group, including reduced GPX4 activity, upregulated levels of 12-HETE and 15-HETE, as well as LPO, LDH, and higher green oxidized fluorescence, compared to the OGD/R + UBIAD1-OE group (Fig. 13G–L).

To investigate the effects of UBIAD1-modulated Golgi apparatus dysfunction on ferroptotic neuronal death, GSH was used as an antioxidant to protect against Golgi apparatus stress [19] and to assess ferroptosis in UBIAD1-siRNA neurons. After GSH treatment, the suppressed CoQ10 levels in the insulted neurons of the OGD/R + UBIAD1-siRNA group were significantly increased in the OGD/R + UBIAD1-siRNA + GSH group (Additional file 5: B). Moreover, Fig. 13M–P shows that compared to the OGD/R + UBIAD1-siRNA group, the effects of downregulated expressions of GPX4 and FTH1 proteins and increased ASCL4 expressions were improved after NAC treatment of the OGD/R + UBIAD1-siRNA + GSH group. Furthermore, GSH treatment significantly reduced Fe^2+^ and total iron levels while improving the levels of lipid peroxidation indices in the OGD/R + UBIAD1-siRNA + GSH group, compared to the OGD/R + UBIAD1-siRNA group (Fig. 13Q–X). Therefore, upregulated UBIAD1 levels remarkably ameliorated oxidative stress by increasing CoQ10 levels and eNOS-mediated NO generation via preventing Golgi apparatus dysfunction, thereby mitigating ferroptotic neuronal death and lipid peroxidation after OGD/R insult.

Collectively, these findings show that UBIAD1 may be a potential neuroprotective agent for reversing mitochondrial and Golgi apparatus impairments, thereby increasing antioxidant capacities and preventing excess formation of lipid peroxides and ferroptotic neuronal death after cerebral ischemic stroke. Moreover, our data suggests that the mitochondria and Golgi apparatus, which are critical subcellular organelles, may be significant treatment targets for ischemic stroke-induced ferroptosis.

## Discussion

In this study, we confirmed the occurrence of ferroptosis and lipid peroxidation after cerebral I/R injury. Both MCAO and OGD/R models exhibited significantly increased 12-HETE and 15-HETE levels. This finding has not been previously reported in ischemic stroke. Furthermore, ferroptosis factors correlated with increasing reperfusion time point duration after 4 h of OGD insult in neurons, with the most noticeable changes in ferroptosis occurring at the reperfusion 12-h time point. This indicated that ferroptotic neuronal death is relatively common at the middle stage of the reperfusion process. Meanwhile, inhibition of ferroptosis and lipid peroxidation prevented brain tissue impairment and neuronal death after cerebral I/R insult. This study provides a potential target for ischemic stroke therapy that could prevent cerebral I/R damage-induced ferroptotic neuronal death.

UBIAD1 is involved in oxidative stress, lipid and cholesterol metabolism via catalyzing CoQ10 biosynthesis in the Golgi apparatus membrane, and maintaining mitochondrial function as well as metabolism through vitamin K2 synthesis [21, 24, 26, 45–47]. However, the significance of UBIAD1 in ferroptotic neuronal death after cerebral I/R insult has not been conclusively determined. We confirmed that UBIAD1 is expressed in cerebral tissues, where it is found in neurons, astrocytes, and microglia. In agreement with previous studies [28, 48, 49], suppressed UBIAD1 levels due to cerebral I/R were noted in both in vivo and vitro models. The upregulated UBIAD1 levels prevented brain tissues and neuronal impairments by inhibiting ferroptosis and lipid peroxidation during cerebral I/R. UBIAD1 knock-down yielded the opposite results. Therefore, UBIAD1 is involved in ferroptotic neuronal death regulation after cerebral I/R.

The mechanisms through which UBIAD1 regulates ferroptotic neuronal death after cerebral I/R, and whether there is a link between UBIAD1-mediated subcellular organelles and ferroptosis following cerebral I/R challenge are unknown. Ferroptosis is induced by dysregulation of homeostasis between the oxidative and antioxidative network. As the central organelle of cellular ROS generation, the mitochondria is involved in modulation of cellular oxidative stress and lipid peroxidation under various I/R events [50, 51]. The mitochondria associated protein (FtMt) was shown to exert protective effects on ferroptosis by modulating iron levels after cerebral ischemic stroke [52]. In this study, we found that UBIAD1 was sub-localized in the mitochondria or in the Golgi apparatus of neurons. Under oxidative stress conditions, the originally sufficient number of mitochondrial cristae and tight matrix, accompanied by normal length and shape of mitochondrial were disrupted. These disruptions included reductions in the number of mitochondrial cristae, hypodense matrix, as well as shorter length and swollen mitochondrial morphologies [35, 53]. Alba et al. indicated that the mitochondrial cristae shape determined mitochondrial metabolism, particularly in mitochondrial respiratory chain functions [54, 55]. We established that upregulated UBIAD1 levels markedly mitigated insulted mitochondrial ultrastructures induced by OGD/R in neurons, thereby improving mitochondrial functions. However, UBIAD1 knockdown revealed contrasting findings. Therefore, it was postulated that the functions of UBIAD1 in biosynthesizing vitamin K2 is involved in mitochondrial respiration by preventing OGD/R-induced reductions in mitochondrial complex activities and restoring mitochondrial functions, thereby enhancing their antioxidative capacities, and eventually leading to ferroptosis and lipid peroxidation inhibitions [18, 25, 45, 47]. The neuroprotective effects of UBIAD1 by enhancing antioxidant capacities through the prevention of mitochondrial dysfunction may serve as a potential mechanism for abolishing ferroptotic neuronal death after cerebral I/R. Consequently, regulation of mitochondrial dysfunctions represents a potential therapeutic strategy for inhibiting ferroptotic neuronal death after ischemic stroke.

The Golgi apparatus, a membrane subcellular organelle, is involved in sorting and processing lipids as well as proteins for cellular secretion [56]. However, the roles and potential mechanisms of Golgi apparatus-mediated ferroptotic neuronal death have not been reported, particularly in cerebral ischemic stroke [14]. Our findings suggest that elevated UBIAD1 levels reversed OGD/R insult-mediated damaging effects on the Golgi apparatus ultrastructure and stress. Further, UBIAD1 regulated ferroptosis and lipid peroxidation by enhancing antioxidant capacities via resolving Golgi apparatus dysfunctions and promoting the generation of non-mitochondrial CoQ10 in injured neurons after OGD/R challenge. Although the mechanism through which Golgi apparatus stress regulates ferroptosis has not been conclusively established, we postulate that reversal of the dysregulated morphology of the Golgi apparatus membrane via UBIAD1 overexpression promoted CoQ10 generation, which alleviated ROS-mediated lipid peroxidation via eNOS-regulated NO generation in insulted neurons after OGD/R [21]. Correspondingly, antioxidant capacities were also enhanced so that the oxidation–reduction imbalance was improved upon I/R induced ferroptotic neuronal death and lipid peroxidation. Bersuker et al. reported that ferroptosis suppressor protein 1 (FSP1), a lipophilic antioxidant, can prevent lipid peroxides formation and suppress ferroptosis by modulating non-mitochondrial CoQ10 levels [22]. Hence, our data confirmed that UBIAD1-mediated upregulation of CoQ10 levels from the Golgi apparatus exerts neuroprotective effects on cerebral I/R-induced ferroptotic neuronal death. Schumacher et al. reported that geranylgeranyl modulates cholesterol metabolism by regulating the transport of UBIAD1 between the endoplasmic reticulum (ER) and the Golgi apparatus [57]. Further, Xu et al. [58] documented that UBIAD1 is in a dynamic equilibrium state between the ER and the Golgi apparatus to modulate cell growth. Therefore, our current data suggested that enhanced non-mitochondrial CoQ10 levels in UBIAD1-overexpression neurons might also play an important role in regulation of mitochondrial dysfunctions upon cerebral I/R-mediated ferroptosis and lipid peroxidation. Moreover, there might be possible crosstalks among ferroptotic neuronal death and lipid peroxidation, mitochondria, and the Golgi apparatus via UBIAD1 during cerebral I/R challenge. Studies should aim at elucidating the dynamic interplays among various subcellular organelles during ferroptotic neuronal death following ischemic stroke.

In conclusion, this study has demonstrated the role of ferroptosis in cerebral I/R insult and confirmed that preventing ferroptotic neuronal death could repair brain tissues and impaired neurons. As a neuroprotective agent, UBIAD1 is involved in I/R-mediated ferroptosis in brain tissues and neurons by enhancing antioxidant capacities via preventing mitochondrial and Golgi apparatus impairments (Fig. 13). Furthermore, prevention of mitochondrial and Golgi apparatus dysfunctions is a possible mechanism for regulating ferroptotic neuronal death during ischemic stroke.

## Materials and methods

### Primary cortical neurons culture

The primary cortical neurons were harvested from 16 to 18 days old Sprague-Dawley (SD) rat embryos following a previously reported protocol [59]. The obtained rat brain tissues were dissected bluntly and then digested with papain for 10 min at 37 °C. Cortical cells were suspended in culture medium containing DMEM and 10% FBS (Gibco, NY, USA) and plated at the bottom of the poly-d-lysine glass dishes (size of 18 mm) at a density of 2 × 10^5^ cells per well in a 12-well plate. After 24 h, the original culture medium was replaced with neurobasal medium containing B27 (Invitrogen, CA, USA), Glutamax (Invitrogen, CA, USA), and 10% FBS. The culture was then incubated at 37 °C and 5% CO_2_.

### Oxygen-glucose deprivation (OGD/R) and reperfusion model

The OGD/R model was performed as described previously [28]. Briefly, to establish ischemic condition, the glucose-free Hanks’ balanced salt solution (D-Hank’s, Biological Industries, Israel) was incubated with neurons and maintained in an anoxic incubator (5% CO_2_ and 95% N_2_) for 4 h at 37 °C after removing the cultured medium of neurons. After 4 h, the insulted neurons were immediately transferred to normal primary culture medium under cultivation conditions (37 °C and 5% CO_2_). Different cultivation time points were used to mimic the reperfusion phase depending on experimental demands.

### Cell transfection of lentiviruses and small interfering RNAs (siRNA)

To achieve UBIAD1 overexpression, primary neurons were transfected with UBIAD1 overexpression lentiviruses and the empty lentivirus vector (Honorgene, Changsha, China) for 72 h, as instructed by the manufacturer. Transfection efficiencies of UBIAD1 overexpression (UBIAD1-OE) and UBIAD1 overexpression vector (vector-UBIAD1-OE) were confirmed by western blot, PCR, and GFP fluorescence assays.

To knock down UBIAD1, primary neurons were transfected with targeting UBIAD1-small interfering RNAs (UBIAD1-siRNA) and non-targeting siRNA (vector-UBIAD1-siRNA) for 24 h as detailed by the manufacturer (Ribobio, Guangzhou, China). Transfection efficiencies of UBIAD1-siRNA and the vector of UBIAD1-siRNA were determined by western blot and PCR assays.

After cell transfections, neurons were divided into the following groups for in vitro assays. UBIAD1-overexpression neurons: (i) CTR + UBIAD1-OE group; (ii) CTR + vector-UBIAD-OE group; (iii) OGD/R + UBIAD1-OE group; and (iv) OGD/R + vector-UBIAD-OE group. UBIAD1-siRNA neurons: (i) CTR + vector-UBIAD-siRNA; (ii) CTR + UBIAD1-siRNA group; (iii) OGD/R + UBIAD1-siRNA group; and (iv) OGD/R + vector-UBIAD1-siRNA group. Each assay was performed at least thrice.

### Establishment of middle cerebral artery occlusion (MCAO) models

The MCAO models were used to mimic cerebral I/R in vivo [59]. The SD rats for model establishment were obtained from WellbioBiotech (Wellbio, Changsha, China). They were anesthetized with about 3% isoflurane and maintained with 1–2% isoflurane. Then, the right common carotid artery (CCA), internal carotid artery (ICA), and external carotid artery (ECA) were surgically exposed. A surgical 4.0 nylon filament was gently inserted to the right of the CCA and slowly advanced into the ICA until the origin of the right middle cerebral artery was occluded. The ischemia phase lasted for 90 min after which the nylon filament was carefully extracted for reperfusion processes. Wounds were gently sutured and the rats allowed to recover from anesthesia. Rats in the sham group received non-inserted nylon filaments, and the remaining surgical operations were similar to those of the MCAO group. Experimental rats were sacrificed after 14 days, and their brain tissues obtained for further assessments.

### Intracranial injection of adeno-associated virus (AAV)

Ipsilateral striatum posterior and cortex in rats were given stereotactic intracranial injections of adeno-associated virus of UBIAD1-overexpression (AAV-UBIAD1-OE) and the empty vector of UBIAD1-overexpression (AAV-vetor-UBIAD1-OE) (Honorgene, Changsha, China) for 21 days before the MCAO surgical procedure, as previously reported [35], whereas the sham group was not injected. Briefly, a flow rate of 0.2 ml/min was used, and 1.04 × 10^10^ gc AAV-UBIAD1-OE and AAV-vector-UBIAD1-OE were injected into the described position below: 10 μl Hamilton microsyringe (Hamilton, Reno, NV, USA) was gently stereotactically inserted into the following coordinate axes: taking the bregma as the origin, medial–lateral (2.5 mm), anterior–posterior (0.2 mm), depth of cortex (2.5 mm), and depth of striatum (4.5 mm). The microsyringe was left in place for 15 min before withdrawing. Transfection efficiencies of AAV-UBIAD1-overexpression in damaged rat brain tissues were determined using GFP fluorescence.

### Drug treatment

To evaluate anti-ferroptosis effects on cerebral I/R in vitro models, the Liproxstain-1 (Lip-1, Selleck, TX, USA) was dissolved to a concentration of 200 nM and applied to neurons for 12 h before OGD/R insult, in line with the manufacturer’s protocol. For the in vivo model, Lip-1 was intraperitoneally (i.p.) administered to rats 24 h before MCAO operation at 10 mg/kg. After pretreatment, SD rats were divided into the following groups. For in vivo experiments: (i) Sham group, (ii) Sham + Lip-1 group, (iii) MCAO group, and (iv) MCAO + Lip-1 group. For in vitro assays: (i) CTR group, (ii) CTR + Lip-1 group, (iii) OGD/R group, and (iv) OGD/R + Lip-1 group.

To determine the role of UBIAD1-mediated mitochondria and the Golgi apparatus in ferroptosis, the oxidative stress-inducing H_2_O_2_ and the anti-oxidant agent, *N*-acetyl cysteine (NAC, Beyond, Shanghai, China) of the mitochondria, as well as the Golgi apparatus stress inducer, brefeldin A (BFA, Sigma-Aldrich, MO, USA) and the anti-Golgi apparatus stress mediator, GSH (Beyond, Shanghai, China) were pretreated for 8 h prior to OGD/R induction. After pretreatment, neurons were divided into the following groups for the in vitro experiments: For mitochondrial investigation: (i) OGD/R + UBIAD1-OE + H_2_O_2_ group, (ii) OGD/R + vector-UBIAD-OE + H_2_O_2_ group, (iii) OGD/R + UBIAD1-siRNA + NAC group, and (iv) OGD/R + vector-UBIAD-siRNA + NAC group. For Golgi apparatus investigation: (i) OGD/R + UBIAD-OE + BFA, (ii) OGD/R + vector-UBIAD1-OE-BFAgroup, (iii) OGD/R + UBIAD1-siRNA + GSH group, and (iv) OGD/R + vector-UBIAD1-siRNA + GSH group. Each experiment was performed at least three times.

### Determination of neurological severity scores

Neurological performance was determined via modified neurological severity scores (mNSS) before the MCAO surgical procedure as well as after 3, 7, 14, and 21 days after MCAO surgical procedure in various experimental groups. The mNSS ranged from 0 to 5, with 0 indicating no neurological deficits and higher scores indicating severe neurologic deficits (more details on mNSS are presented in Additional file 6).

### Immunohistochemistry and immunofluorescence

UBIAD1 protein levels were determined by immunohistochemistry [59]. Briefly, rat brain tissues from different experimental groups were fixed in 10% paraformaldehyde at 4 °C for 24 h. Then, brain tissues were dehydrated and paraffin-embedded prior to being sectioned into 5 μm thick sections. Subsequently, 3% H_2_O_2_ was used to inhibit endogenous peroxidase activities while 10% goat serum was blocked with an antibody buffer and incubated with an anti-UBIAD1 antibody (Abcom, Cambridge, MA). Finally, nuclei were stained with hematoxylin. Immunohistochemistry images were captured and analyzed by fluorescent microscopy (Olympus, Tokyo, Japan) and Image J software (https://imagej.en.softonic.com). Immunofluorescent staining of brain tissues was conducted as previously described. Brain tissue sections were blocked using 10% goat serum followed by overnight incubation with primary target antibodies and for 1 h with secondary antibodies (details of antibodies are given in Additional file 1: Table S1). Fluorescence microscopy (Olympus, Tokyo, Japan) was performed to obtain immunofluorescence images.

### Western blot analysis

Expressions of target proteins were evaluated by western blot assays. Proteins were extracted from lysed neurons and homogenized brain tissues, separated on SDS PAGE, and carefully transferred to PVDF membranes. Membranes were blocked with 5% skim milk and incubated overnight in the presence of primary and secondary antibodies (details of the antibodies are recorded in Additional file 7). Blot images were obtained using an ECL reagent kit (Advansta, Can, USA) and a chemiluminescence system (Bio-Rad, CA, USA).

### Real-time PCR

Total RNA was extracted from neurons or brain tissues using RNA prep Pure Cell Kit (Tiangen, Beijing, China) according to the manufacturer’s protocol. Subsequently, the quantitative real-time PCR (qRT-PCR) was conducted using SYBR Green Master Mix (Qiagen, Shanghai, China). The expression levels of UBIAD1 were then evaluated using a 2^−ΔΔCt^ calculation method.

### TTC staining

After 14 days of reperfusion operation, brain tissues were stained for 15 min at 37 °C using 1% 2,3,5-triphenyltetrazolium chloride (TTC) solution to evaluate infarct sizes of brain tissues after MCAO operation. Non-infarcted brain tissues were identified by red staining, whereas infarct areas were identified by white staining. TTC staining images were analyzed using the Image J software (https://imagej.en.softonic.com).

### Brain water contents

After 14 days of reperfusion, whole rat brains were collected and their wet weights assessed to determine brain water contents after cerebral I/R injury. Then, brain tissues were dried at 100 °C and measured by dry weight. The final brain water content (%) = 100% × (wet brain weight − dry brain weigh)/wet brain weight.

### Hematoxylin–eosin (H&E) staining

To confirm the pathological changes of damaged brain tissues after cerebral I/R challenge, insulted brain tissues were obtained 14 days after reperfusion, fixed in paraffin, embedded, and sliced into 5-μm sections, which were stained with hematoxylin–eosin (H&E) as previously reported [59].

### TUNEL staining

To determine apoptosis levels in brain tissues after cerebral I/R insult, TUNEL staining of brain tissues was performed using the TUNEL kit (Keygen Biotech, Nanjing, China), as detailed by the manufacturer. Positive TUNEL cells were counted in three non-overlapping fields in each experimental group, after which the average number of positive cells was calculated.

### Assessment of GPX4 activities

Levels of GPX4 activities were determined using the GPX4 activity assay kit (CUSABIO, Wuhan, China), as instructed by the manufacturer.

### Iron content

The levels of Fe^2+^ and total iron were determined using the iron assay kit (Abcam, Cambridge, MA), following the manufacturer’s instructions.

### Assessment of 12-HETE and 15-HETE levels

The 12-HETE and 15-HETE levels were assessed using the 12-HETE ELISA kit (Abcam, Cambridge, MA) and the 15-HETE ELISA kit (Abcam, Cambridge, MA), according to the manufacturer’s instructions.

### Lipid peroxidation levels

Lipid peroxidation (LPO) levels in lysate brain tissues and neurons were evaluated using the lipid peroxidation assay kit (Jiancheng, Nanjing, China), according to the manufacturer’s instructions. Furthermore, lipid ROS were assayed in neurons using the live-cell analysis reagent, BODIPY 581/591 C11 staining (Thermo Fisher Scientific, USA) according to the manufacturer’s instructions. Images were obtained by fluorescence microscopy (Olympus, Tokyo, Japan).

### Lactate dehydrogenase (LDH) activities and cell viabilities

The LDH activities were evaluated using the LDH assay kit (Beyond, Shanghai, China) as instructed by the manufacturer. The MTT test kit (Beyond, Shanghai, China) was used to evaluate cell viabilities of neurons. Absorbance at 450 nm was measured using a microplate reader (Thermo Fisher Scientific, USA).

### Transmission electron microscopy (TEM)

Ultrastructural changes of the mitochondria and Golgi apparatus (GA) in neurons were determined by TEM. Sections were prepared and observed by HT7700 electron microscopy (Hitachi, Tokyo Japan) and JEM1400 electron microscopy (JEOL, Tokyo, Japan) for the mitochondria and GA ultrastructures, respectively. Abnormal mitochondrial and GA morphologies were evaluated using the Image J software, which measured various morphological parameters in the mitochondria and GA as previously reported [35, 42].

### Mitochondrial function assay

To determine the functions of the mitochondria in neurons, mitochondria-related indices such as ROS production, mitochondrial membrane potential (MMP, ΔΨm), ATP levels, and GSH levels were evaluated. ROS levels were evaluated using the Reactive Oxygen Species Assay kit (Beyond, Shanghai, China), mitochondrial membrane potential was determined using the JC-1 staining kit (Beyond, Shanghai, China), ATP levels were evaluated using the ATP assay kit (Jiancheng, Nanjing, China), while GSH levels were measured using the GSH assay kit (Jiancheng, Nanjing, China), following the manufacturer’s instructions.

### Mitochondrial respiratory complex activities

Mitochondrial metabolism functions in neurons were quantitatively determined by measuring the activities of mitochondrial respiratory complexes using the mitochondrial complex activity assay kits (Jiancheng A089-1-1 for complex I, Jiancheng A089-2 for complex II, Jiancheng A089-3 for complex III, Jiancheng A089-4-1 for complex IV, and Jiancheng A089-5-1 for complex V).

### Oxidative stress indices

To assess oxidative stress changes in neurons, superoxide dismutase (SOD) levels were determined using the Total Superoxide Dismutase Assay Kit (Beyond, Shanghai, China), malondialdehyde (MDA) levels were assessed using the Lipid Peroxidation MDA Assay Kit (Beyond, Shanghai, China), while total antioxidant capacities (T-AOC) were confirmed using the Total Antioxidant Capacity Assay Kit (Beyond, Shanghai, China), as instructed by the corresponding manufacturers.

### CoQ10 and NO levels

The CoQ10 detection kit (Leagene, Beijing, China) was used to assess CoQ10 levels. Absorbance at 620 nm was measured using a microplate reader (Thermo Fisher Scientific, MA, USA). The NO levels were assessed using the NO Assay Kit (Beyond, Shanghai, China), as instructed by the manufacturer. Absorbance at 560 nm was measured using a microplate reader (Thermo Fisher Scientific, MA, USA).

### Statistical analyses

The GraphPad Prism 9.0 software (https://www.graphpad.com) was used for statistical analyses. The Kolmogorov–Smirnov Test was used to assess data distribution. Subsequently, data were analyzed using a two-tailed Students’ t test for two independent variables while the two-way repeated-measures analysis of variance (ANOVA) with Tukey’s post hoc test was used to analyze neurologic severity scores (mNSS score). Comparisons of means among three or more groups was done by one-way ANOVA or two-way ANOVA followed by the Tukey’s post hoc test. All data are presented as means ± SD. Differences between mean values were considered significant at p < 0.05.

## Supplementary Information

**Additional file 1.** The quantification of the changes of mitochondrial morphology in neurons.

**Additional file 2.** The levels of ROS production in neurons.

**Additional file 3.** The levels of oxidative stress in H_2_O_2_ and NAC treatment neurons.

**Additional file 4.** The quantification of the alteration of Golgi apparatus morphology in neurons.

**Additional file 5.** The levels of CoQ10 generation in BFA and GSH treatment neurons.

**Additional file 6.** Modified neurological severity score grading system.**Additional file 7.** Primary and secondary antibodies.

**Additional file 7.** Primary and secondary antibodies.

## Data Availability

The authors declare that all data supporting the findings of this study are available within the paper and its Additional information files.

## Abbreviations

I/R: Ischemic/reperfusion
CoQ10: Coenzyme Q10
eNOS: Endothelial nitric oxide synthase
NO: Nitric oxide
GSH: Glutathione
BFA: Brefeldin A
ARF1: Associated with small G protein
SCD: Schnyder corneal dystrophy
OGD/R: Oxygen-glucose deprivation (OGD/R) and reperfusion
UBIAD1-OE: UBIAD1-overexpression
UBIAD1-siRNA: UBIAD1-small interfering RNAs
GPX4: Glutathione peroxidase 4
ER: Endoplasmic reticulum
MCAO: Middle cerebral artery occlusion
CCA: Common carotid artery
ICA: Internal carotid artery
ECA: External carotid artery
SD rat: Sprague-Dawley rat
AAV: Adeno-associated virus
MDA: Malondialdehyde
LPO: Lactoperoxidase
Lip-1: Liproxstain-1
NeuN: Neuron NeuN
GFAP: Astrocytes
Iba-1: Microglia
LDH: Lactate dehydrogenase
mNSS: Modified neurological severity scores
TTC: Triphenyltetrazolium chloride
HE: Hematoxylin–eosin
TEM: Transmission electron microscopy
MMP, ΔΨm: Mitochondrial membrane potential
SOD: Superoxide dismutase
T-AOC: Total antioxidant capacity
GA: Golgi apparatus
Mito: Mitochondria
