# Supplementary material for: UBIAD1 alleviates ferroptotic neuronal death by enhancing antioxidative capacity by cooperatively restoring impaired mitochondria and Golgi apparatus upon cerebral ischemic/reperfusion insult
Source: Cell Biosci. 2022 Apr 4;12:42. doi: 10.1186/s13578-022-00776-9 (PMC8981649; doi:10.1186/s13578-022-00776-9)
Supplement: Supplementary file 1 — Additional file 1. The quantification of the changes of mitochondrial morphology in neurons. [file 13578_2022_776_MOESM1_ESM.docx]

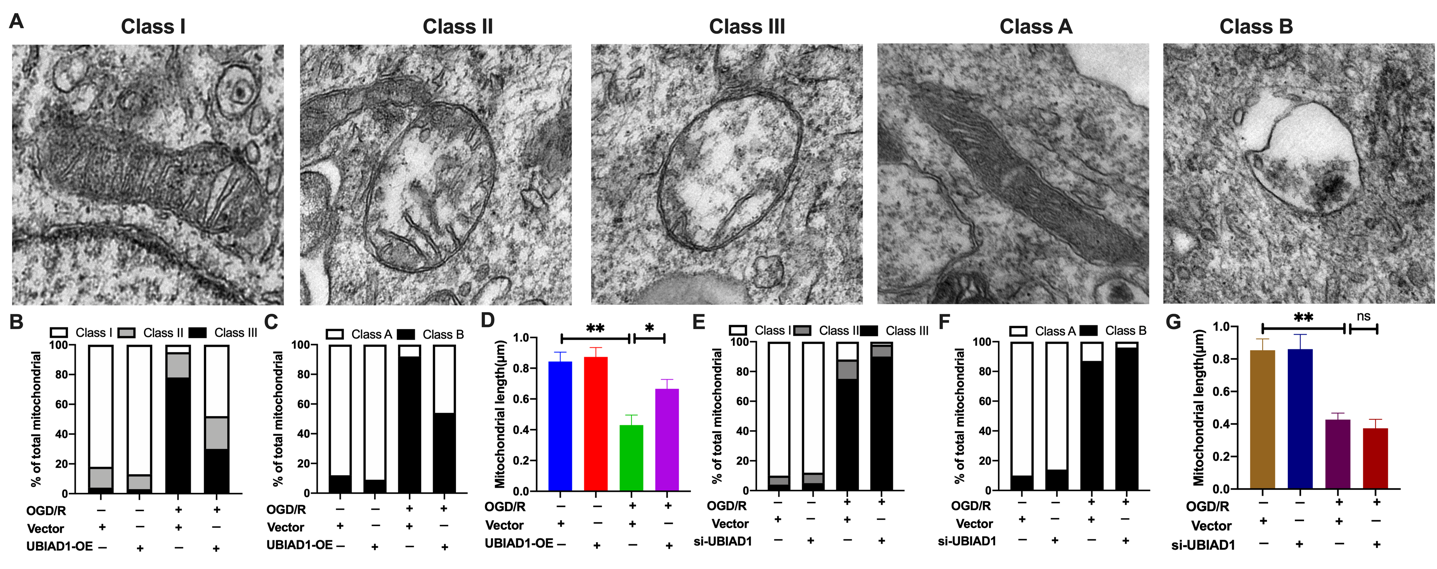


**Additional file 1.** The quantification of the changes of mitochondrial morphology in neurons. **A** The number of mitochondrial cristae, matrix density, and level of swelling in neurons as assessed using TEM. **B** and **E** Mitochondrial cristae in approximately 60 mitochondria per group were evaluated using three grade criteria (Class I: more than four cristae, Class II: two to three cristae, Class III: less than one cristae). **C** and **F** The matrix density of mitochondria in approximately 60 mitochondria per group as recorded using two grades criteria (Class A: dense matrix in mitochondria and Class B: hypodense matrix in swollen mitochondria). **D** and **G** The length of mitochondria in approximately 60 mitochondria per group. All the data are expressed as the mean ± SD, *P ＜ 0.05, ***P* ＜ 0.01; OGD/R + vector-UBIAD1-OE group compared to OGD/R + UBIAD1-OE group or CTR + vector + UBIAD1-OE group. OGD/R + vector-UBIAD1-siRNA group compared to OGD/R + UBIAD1-siRNA group or CTR + vector + UBIAD1-siRNA group.
