# Supplementary material for: UBIAD1 alleviates ferroptotic neuronal death by enhancing antioxidative capacity by cooperatively restoring impaired mitochondria and Golgi apparatus upon cerebral ischemic/reperfusion insult
Source: Cell Biosci. 2022 Apr 4;12:42. doi: 10.1186/s13578-022-00776-9 (PMC8981649; doi:10.1186/s13578-022-00776-9)
Supplement: Supplementary file 2 — Additional file 2. The levels of ROS production in neurons. [file 13578_2022_776_MOESM2_ESM.docx]

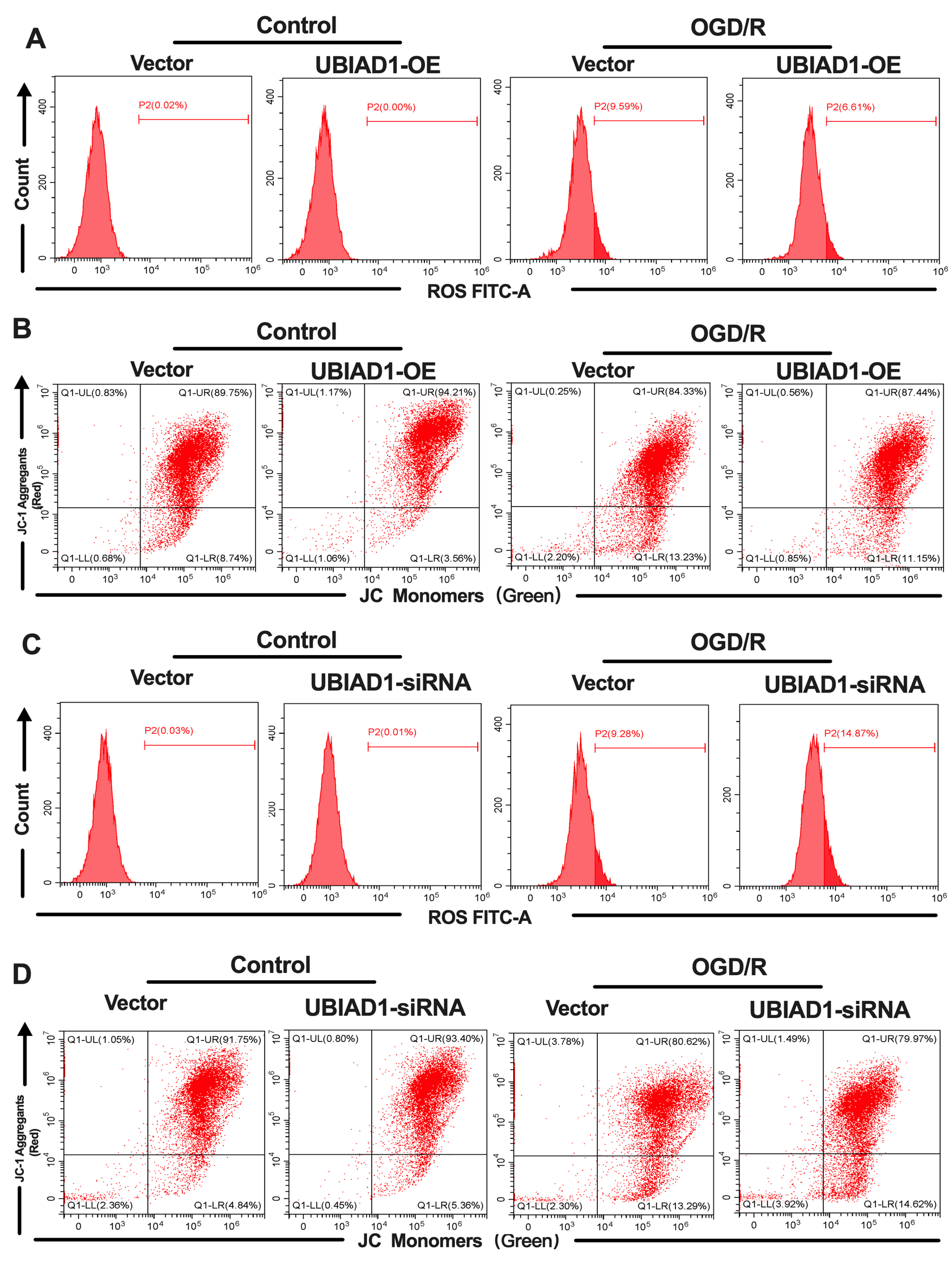


**Additional file 2.** The levels of ROS production in neurons. **A** The ROS production was confirmed by DCFH-DA assay in UBIAD1 overexpression groups. **B** The level of mitochondrial membrane potential was determined by JC-1 staining in UBIAD1 overexpression groups. **C** The ROS production was confirmed by DCFH-DA assay in the knock-down of UBIAD1 groups. **D** The level of mitochondrial membrane potential was determined by JC-1 staining in the knock-down of UBIAD1 groups. All data are presented as the mean value ±SD (n=3). Compared with the control group: **P<0.01; Compared with the control group and vector group: *P<0.05, **P<0.01. OGD/R+vector-UBIAD1-OE group relative to OGD/R+UBIAD1-OE group or CTR+vector+UBIAD1-OE group. OGD/R+vector-UBIAD1-siRNA group compared to OGD/R+UBIAD1-siRNA group or CTR+vector+UBIAD1-siRNA group.
