# Supplementary material for: UBIAD1 alleviates ferroptotic neuronal death by enhancing antioxidative capacity by cooperatively restoring impaired mitochondria and Golgi apparatus upon cerebral ischemic/reperfusion insult
Source: Cell Biosci. 2022 Apr 4;12:42. doi: 10.1186/s13578-022-00776-9 (PMC8981649; doi:10.1186/s13578-022-00776-9)
Supplement: Supplementary file 3 — Additional file 3. The levels of oxidative stress in H2O2 and NAC treatment neurons. [file 13578_2022_776_MOESM3_ESM.docx]

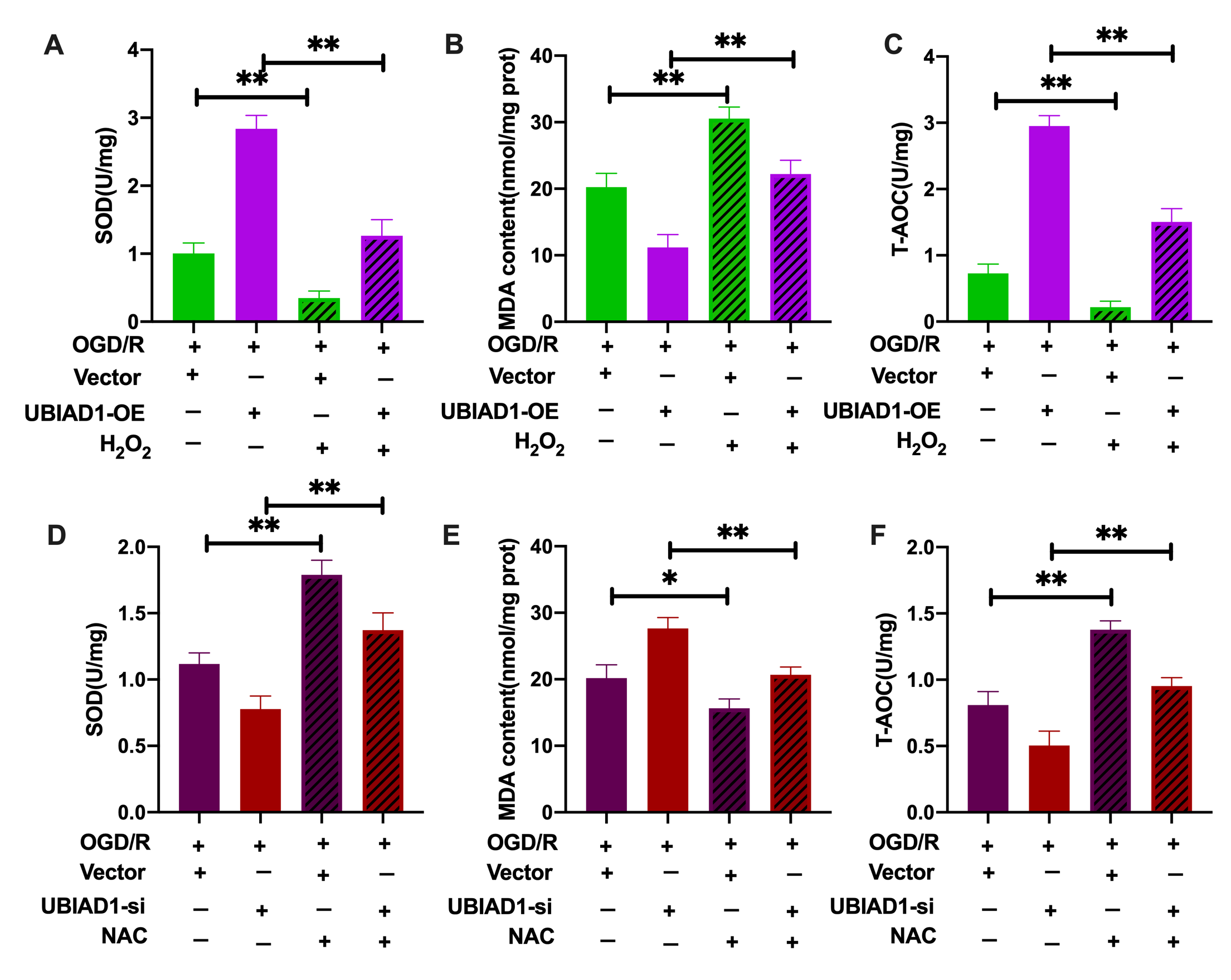


**Additional file 3.**The levels of oxidative stress in H_2_O_2_ and NAC treatment neurons. **A** and **D** The change of SOD production was evaluated by WST-8 assay kit in various experimental groups. **B** and **E** The level of MAD generation was determined by lipid peroxidation assay kit in various experimental groups. **C** and **F** The level of T-AOC was defined by ABTS assay kit in various experimental groups. All the data are expressed as the mean±SD, *P＜0.05，**P＜0.01；OGD/R+vector-UBIAD1-OE group relative to OGD/R+UBIAD1-OE group or CTR+vector+UBIAD1-OE group. OGD/R+vector-UBIAD1-siRNA group compared to OGD/R+UBIAD1-siRNA group or CTR+vector+UBIAD1-siRNA group.
