# Supplementary material for: UBIAD1 alleviates ferroptotic neuronal death by enhancing antioxidative capacity by cooperatively restoring impaired mitochondria and Golgi apparatus upon cerebral ischemic/reperfusion insult
Source: Cell Biosci. 2022 Apr 4;12:42. doi: 10.1186/s13578-022-00776-9 (PMC8981649; doi:10.1186/s13578-022-00776-9)
Supplement: Supplementary file 4 — Additional file 4. The quantification of the alteration of Golgi apparatus morphology in neurons. [file 13578_2022_776_MOESM4_ESM.docx]

**
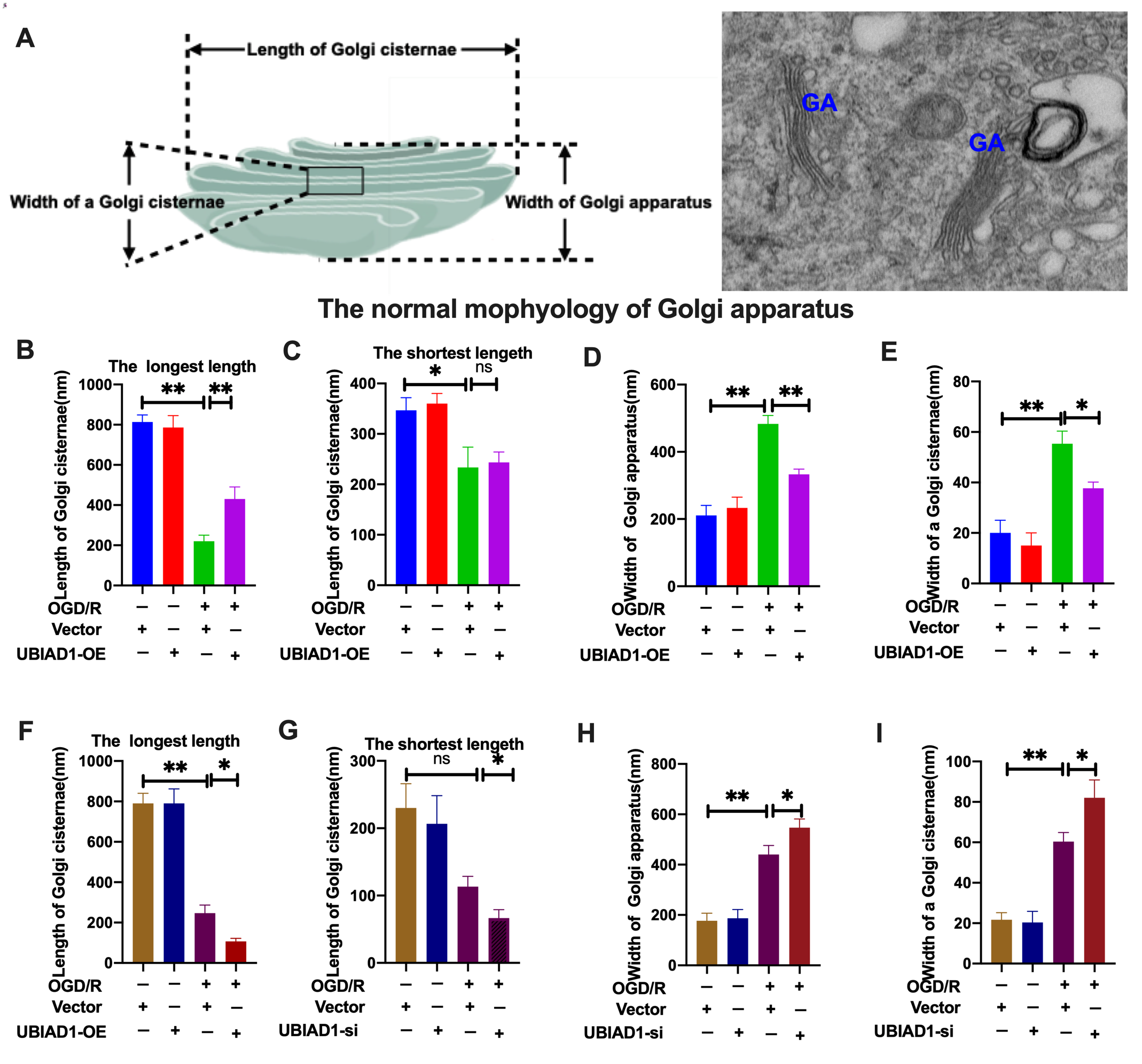
**

**Additional file 4.**The quantification of the alteration of Golgi apparatus morphology in neurons. **A** Images showing the normal morphology of Golgi apparatus. **B-C** and **F-G** Assessment of the length of Golgi cisternae in approximately 30 Golgi apparatus per group. **D** and **H** The width of Golgi apparatus in nearly 30 Golgi apparatus per group. **E** and **I** The width of a Golgi cisternae in nearly 30 Golgi apparatus per group. All the data are expressed as the mean ± SD, **P* ＜ 0.05，***P* ＜ 0.01; OGD/R + vector-UBIAD1-OE group relative to OGD/R + UBIAD1-OE group or CTR + vector + UBIAD1-OE group. OGD/R + vector-UBIAD1-siRNA group compared to OGD/R + UBIAD1-siRNA group or CTR + vector + UBIAD1-siRNA group.
