# Supplementary material for: UBIAD1 alleviates ferroptotic neuronal death by enhancing antioxidative capacity by cooperatively restoring impaired mitochondria and Golgi apparatus upon cerebral ischemic/reperfusion insult
Source: Cell Biosci. 2022 Apr 4;12:42. doi: 10.1186/s13578-022-00776-9 (PMC8981649; doi:10.1186/s13578-022-00776-9)
Supplement: Supplementary file 5 — Additional file 5. The levels of CoQ10 generation in BFA and GSH treatment neurons. [file 13578_2022_776_MOESM5_ESM.docx]

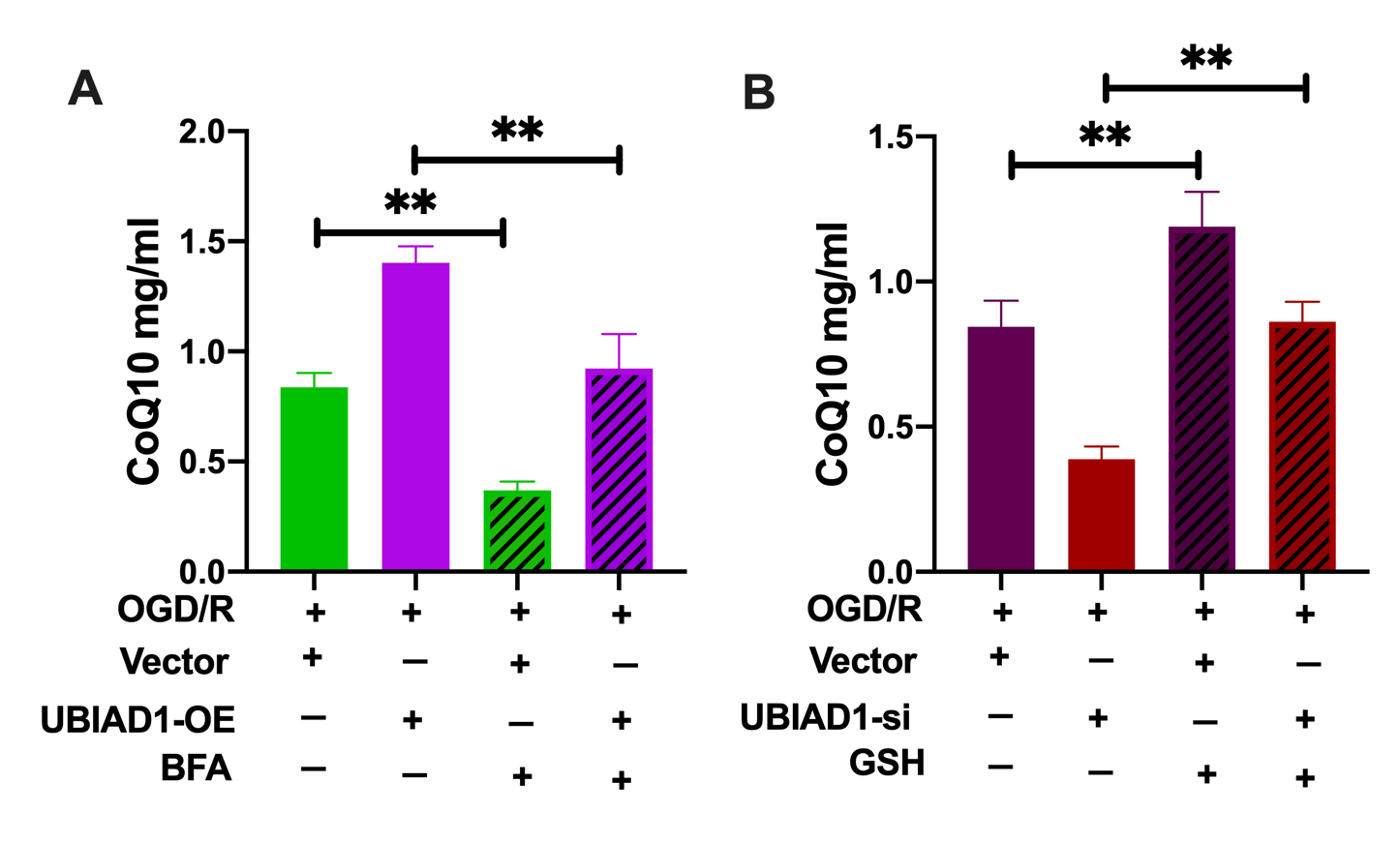


**Additional file 5.** The levels of CoQ10 generation in BFA and GSH treatment neurons. **A** The level of CoQ10 production was evaluated by CoQ10 assay kit in UBIAD1 overexpression groups. **B** The change of CoQ10 generation was evaluated by CoQ10 assay kit in UBIAD1-siRNA groups. All the data are expressed as the mean±SD, *P＜0.05，**P＜0.01；OGD/R+vector-UBIAD1-OE group relative to OGD/R+UBIAD1-OE group or CTR+vector+UBIAD1-OE group. OGD/R+vector-UBIAD1-siRNA group compared to OGD/R+UBIAD1-siRNA group or CTR+vector+UBIAD1-siRNA group.
