# Supplementary material for: UBIAD1 alleviates ferroptotic neuronal death by enhancing antioxidative capacity by cooperatively restoring impaired mitochondria and Golgi apparatus upon cerebral ischemic/reperfusion insult
Source: Cell Biosci. 2022 Apr 4;12:42. doi: 10.1186/s13578-022-00776-9 (PMC8981649; doi:10.1186/s13578-022-00776-9)
Supplement: Supplementary file 6 — Additional file 6. Modified neurological severity score grading system.Additional file 7. Primary and secondary antibodies. [file 13578_2022_776_MOESM6_ESM.docx]

**Additional file 6.** Modified neurological severity score grading system.

| Score grading | | |
| --- | --- | --- |
|  | | |
| 0 | Normal |  |
| 1-6 | Mild injury |  |
| 7-12 | Moderate injury |  |
| 13-18 | Serious damage |  |
| 18 | Most severe neurological deficits |  |
