# Supplementary material for: UBIAD1 alleviates ferroptotic neuronal death by enhancing antioxidative capacity by cooperatively restoring impaired mitochondria and Golgi apparatus upon cerebral ischemic/reperfusion insult
Source: Cell Biosci. 2022 Apr 4;12:42. doi: 10.1186/s13578-022-00776-9 (PMC8981649; doi:10.1186/s13578-022-00776-9)
Supplement: Supplementary file 7 — Additional file 7. Primary and secondary antibodies. [file 13578_2022_776_MOESM7_ESM.docx]

**Additional file 7.** Primary and secondary antibodies.

| **Primary antibodies** | | | | |
| --- | --- | --- | --- | --- |
| Product name | Catalogue Number | Host species | Concentration | Supplier |
| GP4X | 14432-1-AP | Rabbit | 1：750 | Proteintech |
| FTH1 | ab65080 | Rabbit | 1ug/ml | Abcam |
| ACSL4 | 22401-1-AP | Rabbit | 1：1000 | Proteintech |
| UBIAD1 | ab191691 | Rabbit | 2ug/ml | Abcam |
| GM130 | 11308-1-AP | Rabbit | 1：5000 | Proteintech |
| GOLPH3 | ab91492 | Rabbit | 1ug/ml | Abcam |
| SPCA1 | ab126171 | Rabbit | 1：1000 | Abcam |
| β-actin | 60008-1-Ig | Mouse | 1：5000 | Proteintech |
| **Secondary antibodies** | | | | |
| HRP goat anti-mouse IgG | SA00001-1 |  | 1：5000 | Proteintech |
| HRP goat anti-rabbit IgG | SA00001-2 |  | 1：6000 | Proteintech |
